# Integrative clustering reveals a novel split in the luminal A subtype of breast cancer with impact on outcome

**DOI:** 10.1186/s13058-017-0812-y

**Published:** 2017-03-29

**Authors:** Miriam Ragle Aure, Valeria Vitelli, Sandra Jernström, Surendra Kumar, Marit Krohn, Eldri U. Due, Tonje Husby Haukaas, Suvi-Katri Leivonen, Hans Kristian Moen Vollan, Torben Lüders, Einar Rødland, Charles J. Vaske, Wei Zhao, Elen K. Møller, Silje Nord, Guro F. Giskeødegård, Tone Frost Bathen, Carlos Caldas, Trine Tramm, Jan Alsner, Jens Overgaard, Jürgen Geisler, Ida R. K. Bukholm, Bjørn Naume, Ellen Schlichting, Torill Sauer, Gordon B. Mills, Rolf Kåresen, Gunhild M. Mælandsmo, Ole Christian Lingjærde, Arnoldo Frigessi, Vessela N. Kristensen, Anne-Lise Børresen-Dale, Kristine K. Sahlberg, OSBREAC, Elin Borgen, Olav Engebråten, Øystein Fodstad, Britt Fritzman, Øystein Garred, Gry A. Geitvik, Solveig Hofvind, Hege G. Russnes, Helle Kristine Skjerven, Therese Sørlie

**Affiliations:** 1 Department of Cancer Genetics, Institute for Cancer Research, Oslo University Hospital, The Norwegian Radium Hospital, Oslo, Norway; 2 K.G. Jebsen Centre for Breast Cancer Research, Institute for Clinical Medicine, University of Oslo, Oslo, Norway; 3 Oslo Center for Biostatistics and Epidemiology, Institute of Basic Medical Science, University of Oslo, Oslo, Norway; 4 Department of Clinical Molecular Biology (EpiGen), Division of Medicine, Akershus University Hospital, Lørenskog, Norway; 5 Department of Circulation and Medical Imaging, Norwegian University of Science and Technology (NTNU), Trondheim, Norway; 6 Genome-Scale Biology Research Program, University of Helsinki, Helsinki, Finland; 7 Department of Tumor Biology, Institute for Cancer Research, Oslo University Hospital, The Norwegian Radium Hospital, Oslo, Norway; 8 Five3 Genomics, LLC, Santa Cruz, CA 95060 USA; 9 Department of Systems Biology, University of Texas M.D. Anderson Cancer Center, Houston, TX USA; 10 Cambridge University Hospitals Trust, Addenbrookes Hospital, Cambridge, UK; 11 Cancer Research UK Cambridge Institute, University of Cambridge, Cambridge, UK; 12 Department of Experimental Clinical Oncology, Aarhus University Hospital, Aarhus, Denmark; 13 Department of Oncology, Akershus University Hospital, Lørenskog, Norway; 14 Institute of Clinical Medicine, Faculty of Medicine, University of Oslo, Oslo, Norway; 15 Department of Surgery, Akershus University Hospital, Lørenskog, Norway; 16 The Oslo Breast Cancer Research Consortium (OSBREAC) www.osbreac.no, Oslo, Norway; 17 Department of Oncology, Division of Cancer Medicine, Oslo University Hospital, Oslo, Norway; 18 Department of Breast and Endocrine Surgery, Oslo University Hospital, Oslo, Norway; 19 Department of Pathology, Akershus University Hospital, Lørenskog, Norway; 20 Centre for Cancer Biomedicine, University of Oslo, Oslo, Norway; 21 Department of Computer Science, University of Oslo, Oslo, Norway; 22 Oslo Center for Biostatistics and Epidemiology, Oslo University Hospital, Oslo, Norway; 23 Department of Research, Vestre Viken Hospital Trust, Drammen, Norway

**Keywords:** Breast cancer, Integration, Luminal A, Consensus clustering, MicroRNA

## Abstract

**Background:**

Breast cancer is a heterogeneous disease at the clinical and molecular level. In this study we integrate classifications extracted from five different molecular levels in order to identify integrated subtypes.

**Methods:**

Tumor tissue from 425 patients with primary breast cancer from the Oslo2 study was cut and blended, and divided into fractions for DNA, RNA and protein isolation and metabolomics, allowing the acquisition of representative and comparable molecular data. Patients were stratified into groups based on their tumor characteristics from five different molecular levels, using various clustering methods. Finally, all previously identified and newly determined subgroups were combined in a multilevel classification using a “cluster-of-clusters” approach with consensus clustering.

**Results:**

Based on DNA copy number data, tumors were categorized into three groups according to the complex arm aberration index. mRNA expression profiles divided tumors into five molecular subgroups according to PAM50 subtyping, and clustering based on microRNA expression revealed four subgroups. Reverse-phase protein array data divided tumors into five subgroups. Hierarchical clustering of tumor metabolic profiles revealed three clusters. Combining DNA copy number and mRNA expression classified tumors into seven clusters based on pathway activity levels, and tumors were classified into ten subtypes using integrative clustering. The final consensus clustering that incorporated all aforementioned subtypes revealed six major groups. Five corresponded well with the mRNA subtypes, while a sixth group resulted from a split of the luminal A subtype; these tumors belonged to distinct microRNA clusters. Gain-of-function studies using MCF-7 cells showed that microRNAs differentially expressed between the luminal A clusters were important for cancer cell survival. These microRNAs were used to validate the split in luminal A tumors in four independent breast cancer cohorts. In two cohorts the microRNAs divided tumors into subgroups with significantly different outcomes, and in another a trend was observed.

**Conclusions:**

The six integrated subtypes identified confirm the heterogeneity of breast cancer and show that finer subdivisions of subtypes are evident. Increasing knowledge of the heterogeneity of the luminal A subtype may add pivotal information to guide therapeutic choices, evidently bringing us closer to improved treatment for this largest subgroup of breast cancer.

**Electronic supplementary material:**

The online version of this article (doi:10.1186/s13058-017-0812-y) contains supplementary material, which is available to authorized users.

## Background

Breast cancer is a disease that has been thoroughly profiled on various levels revealing heterogeneity that is manifest at the clinical, histopathological and molecular level. At each level, separation of breast tumors into different groups has been used to identify subgroups of the disease, which assists patient management. The two major groups of breast cancer at the histopathological level are the estrogen receptor (ER)-positive and ER-negative tumors, encompassing all other molecular subgroups. Further, at the gene expression level, five main subgroups have been identified [1, 2], and combining gene expression with copy number data further refined breast cancer into 10 integrated subgroups with different genomic and transcriptomic profiles and prognosis [3].

Recently, mutation data coupled to these 10 subgroups showed how functional mutations in the *PIK3CA* gene were associated with different survival times in ER-positive breast cancer when stratifying by these integrated subgroups [4]. Integrating classifications extracted from four different levels (mRNA, microRNA (miRNA) expression, DNA copy number and methylation) revealed new insights into the biology and immune profile of pre-invasive and invasive breast cancers [5], while metabolic analyses have revealed three naturally occurring clusters with distinct metabolic profiles [6]. Exploring the causes and consequences of breast cancer at a higher level may lead to refined therapeutic strategies.

Tumor development and progression is a dynamic evolutionary process involving genomic and epigenetic aberrations, cellular context, influence from the surrounding environment and patient-specific characteristics. Furthermore, cancer is increasingly being understood as a disease with alterations at the network level where multiple different changes can engender a similar cancer phenotype or outcome [7]. Integration of molecular data is needed to uncover these alterations in single tumors and further link them across patients to understand the effects on network levels. Also, integrative analyses may generate explanatory power that one data type alone cannot provide [8]. The long-term goal of this approach is further stratification of patients into subgroups for improved tailored therapy. The information content in integrated analyses is higher than in any of the separate molecular-level studies; however, the availability of all these layers of data from the same patients is often limited.

Using data from five molecular platforms (mRNA expression, protein expression, miRNA expression, DNA copy number and methylation), The Cancer Genome Atlas (TCGA) performed a multiplatform integrative analysis on 348 breast tumors [9]. The subtypes (clusters) defined from each of the molecular levels were subjected to unsupervised consensus clustering revealing four major patient groups. These “higher-order” subtypes corresponded well with the mRNA expression-defined PAM50 subtypes and as such did not identify new subgroups within the subtypes. The same cluster-of-clusters approach was also applied to the corresponding molecular data from 12 different cancer types [10], revealing 11 major subtypes. Interestingly, although most of the multiplatform subtypes correlated with tissue of origin, some of the tumor types coalesced into one subtype, while, for example, breast cancer was split into two subtypes and bladder cancer into three different subtypes [10].

The Oslo2 study is a multicenter study initiated in 2006, in which patients with breast cancer were enrolled from Oslo University Hospital. So far, 2000 patients have been enrolled into the study, and here we present an analysis of the first 355 patients in addition to 70 patients from a similar study performed at Akershus University Hospital. In this study, we integrated seven different classifications extracted from five molecular levels; DNA copy number, mRNA, miRNA and protein expression and tumor metabolic profiles. The aim was to identify higher-order integrated subtypes. Whenever possible, we used existing clustering schemes, as developed and tested in the literature, including the cluster-of-clusters analysis (COCA) methods previously described [9, 10]. In this way we provided comparable evidence on how the population-based Oslo2 cohort represents the previously described subtypes and on how these compare to newly identified subtypes.

## Methods

### The Oslo2 clinical cohort

Oslo2 is a multicenter study in which patients with primary operable breast cancer (cancer tumor stage (cT)1–cT2) were consecutively enrolled at Oslo University Hospital, Norway (including the Radium Hospital and Ullevål Hospital, Vestre Viken and Østfold Hospital in southeast Norway). Patients were included from 2006, at the time of primary surgery after giving written informed consent. Here we present an analysis of the first subset of 355 patients. The Regional Committee for Medical and Health Research Ethics for southeast Norway has approved the study (approval number 1.2006.1607 and 1.2007.1125, 2009/615, 2009/4935).

Experienced breast pathologists macroscopically evaluated the surgical specimens before parts of the tumor were fresh frozen (-80 °C). Patients were followed according to national guidelines for follow up after breast cancer treatment. A fresh-frozen biopsy sample from the primary tumor and peripheral blood samples were collected at the time of surgery. In addition, bone marrow and lymph nodes were collected. Tumor tissue and blood specimens were collected from patients who experienced relapse.

Samples from the Oslo2 study were coupled with tumor tissue collected from 70 patients from a similar study conducted at the Akershus University Hospital, Norway (approval number 429-04148) from 2003 to 2010. In total, tumor tissue from 425 patients was used in the current analyses and the patient cohort is collectively described as the Oslo2 cohort.

### Clinical data

Clinical parameters were collected from patient records and from pathology reports. Hormone receptor status for estrogen receptor (ER) and progesterone receptor (PR) were obtained by standard immunohistochemical assessment (IHC). Amplification of the human epidermal growth factor receptor 2 (*HER2*) gene was assessed by a combination of IHC and chromogenic *in situ* hybridization (CISH) following standard guidelines. Experienced pathologists assessed tumor size, morphology, histological grade and axillary lymph node involvement as part of standard diagnostic routine. Age, mode of detection, surgical procedure and presence of metastatic disease at the time of diagnosis were collected from hospital records. Following national guidelines for breast cancer, screening for metastases is not standard procedure in asymptomatic patients at diagnosis. Thus, most patients did not undergo magnetic resonance imaging (MRI) or computed tomography (CT) to detect metastases.

### Tumor preparation

Biopsies from Oslo University Hospital were taken at the time of surgery and fresh-frozen. The tumor was cut into three pieces. Frozen sections were taken from the flanking pieces facing the middle piece, stained with hematoxylin and eosin and evaluated by a pathologist for the presence of tumor cell percentage. The average tumor cell percentage was 53% (range 0–90%). A tumor tissue sample from the middle piece was taken for high-resolution magic-angle spinning magnetic resonance spectroscopy (HR MAS MRS). Following this, the three tumor pieces were merged, cut into smaller pieces by scalpel, mixed and split into dedicated vials for DNA, RNA and protein isolation. Biopsies from Akershus University Hospital were first put on RNAlater (Thermo Fisher scientific, Waltham, MA, USA) overnight before being frozen at -80 °C. The preparation was performed as described previously, but without dedicated vials for HR MAS MRS and protein isolation.

### DNA and RNA isolation

DNA isolation was performed using the Maxwell® 16 instrument (Promega, Fitchburg, WI, USA) and the Maxwell® 16 tissue DNA Purification Kit (Promega). DNA was isolated according to the manufacturer’s protocol. In brief, tumor tissue was transferred into the Maxwell cartridge cassettes predispensed with magnetic beads, lysis buffer, and wash buffers of isopropanol and ethanol. The isolation procedure is automated, starting with sample lysis and tissue homogenization, following bead isolation of DNA, and finally washing steps. The DNA was eluted in 200–600 ul TE-buffer (pH 8.5). DNA was stored at -20 °C.

Total RNA was isolated by phenol-chloroform extraction using the TRIzol reagent (Invitrogen, Carlsbad, CA, USA) following the manufacturer’s instructions and has been described previously [11]. The NanoDrop spectrophotometer (Thermo Fisher scientific) was used to assess the concentration and RNA purity by measuring absorbance at different wavelengths. The quality and integrity of the RNA was assessed by chip‐based capillary electrophoresis using a 2100 Bioanalyzer instrument (Agilent Technologies, Santa Clara, CA, USA). The resulting average RNA integrity number (RIN) of all samples was 5.6, range 1.0–9.7.

### *TP53* sequencing

*TP53* mutation analysis of exon 2-11 was performed by Sanger sequencing using the 3730 DNA Analyzer (Applied Biosystems, Life Technologies Corporation, Carlsbad, CA, USA). PCR amplification with the BigDye Direct Cycle Sequencing Kit (Applied Biosystems) was performed using 5 ng tumor DNA, followed by BigDye XTerminator Purification Kit (Applied Biosystems). The sequences were read in SeqScape v.2.7 (Applied Biosystems) by two independent investigators.

### *PIK3CA* mutation detection

Mutations in the *PIK3CA* gene were detected using a mass spectroscopy-based approach in addition to Sanger sequencing. In total, 314 tumors were evaluated for ten known *PIK3CA* mutations using the Sequenom MassArray MALDI-TOF MassArray system (Sequenom, San Diego, CA, USA) as previously described [12]: *PIK3CA*_C420R_T1258C, *PIK3CA*_E110K_G328A, *PIK3CA*_E542KQ_G1624AC *PIK3CA*_E545KQ_G1633AC, *PIK3CA*_G1049R_G3145C, *PIK3CA*_H1047RL_A3140GT *PIK3CA*_K111N_G333C, *PIK3CA*_N345K_T1035A, *PIK3CA*_P539R_C1616G and *PIK3CA*_Q546LPR_A1637TCG.

A subset of the tumor samples (*n* = 275) were sequenced for detection of mutations in *PIK3CA* exon 9 and 20. PCR touchdown reaction with HotStarTaq DNA polymerase (Qiagen, Hilden, Germany) was performed using 10 ng of DNA. The PCR products were visualized on a 1.5% agarose gel, and the products were cleaned with EpMotion 5075 (Eppendorf AG, Hamburg, Germany). For the sequencing reactions, 3 ul of the purified PCR product and BigDye Terminator v1.1 reaction mix was used. Sequencing reactions were performed on MJ Research Tetrad DNA Engine (MJ Research, Bio-Rad Laboratories Inc., Hercules, CA, USA), and cleaned on Sephadex mini-columns (GE Healthcare Life Sciences, Little Chalfont, UK). Sequencing was performed using a 3730 DNA Analyzer (Applied Biosystems). Mutation scoring was performed in SeqScape v.2.7 (Applied Biosystems) by two independent investigators. For tumor samples evaluated by both approaches, the results were combined by identifying a tumor sample as *PIK3CA*-mutated if at least one of the methods detected a mutation.

### Copy number aberration analysis, segmentation and complex arm aberration index

Tumor DNA was hybridized to Affymetrix SNP 6.0 arrays (Affymetrix, Santa Clara, CA, USA) at Aros Applied Biotechnology (Aarhus, Denmark) following the manufacturer’s recommendations. Tumor samples collected at the Akershus University Hospital were stored on RNAlater. DNA extracted from a majority of these samples did not pass the quality control assessment for single nucleotide polymorphism (SNP) arrays. Affymetrix CEL-files were processed using the PennCNV-Affy library [13], with the HapMap samples as the reference set [14]. Correction for GC-related binding bias was performed [15]. The resulting GC-adjusted LogRs were segmented into regions of constant copy number using the R package “copynumber” [16] in the Comprehensive R Archive Network (CRAN) [17]. The complex arm aberration index (CAAI) was computed as described previously [18]. Note that in the original paper CAAI-values were thresholded only at the value 0.5 to produce a dichotomous variable, but here we also distinguished between the number of arms with a CAAI event; zero arms, one arm or at least two arms.

### Gene expression and PAM50 subtypes

mRNA expression was measured using SurePrint G3 Human GE 8x60K one-color microarrays from Agilent (Agilent Technologies) according to the manufacturer’s protocol and using 100 ng of RNA as input for amplification. The array includes 42,405 unique 60-mer probes, targeting 27,958 Entrez genes and 7419 long intergenic non-coding RNAs (lincRNAs). Scanning was performed with Agilent Scanner G2565A, and signals were extracted using Feature Extraction v.10.7.3.1 (Agilent Technologies). Non-uniform spots were excluded and missing data were imputed using local least squares imputation (LLSimpute from the R package “pcaMethods” [19]). Arrays were log_2_-transformed, quantile-normalized and hospital-adjusted by subtracting from each probe value the mean probe value of the samples from that same hospital. To have a single expression value per gene per sample, the values corresponding to probes with identical Entrez ID were averaged. A cutoff was applied on the RIN value to exclude samples with an RIN value below 2.5. mRNA expression data have been submitted to the Gene Expression Omnibus (GEO) database [GEO:GSE80999].

The PAM50 subtype algorithm [20] was used to assign a gene expression subtype label to each sample. For each sample, a 50-dimensional vector was found by extracting the gene expression values for the 50 genes in the PAM50 gene list. A 50-dimensional centroid vector was then calculated by averaging the gene expression vectors for all the ER-positive samples, and likewise a 50-dimensional centroid vector was calculated by averaging the gene expression vectors for all the ER-negative samples. A combined centroid was then defined as a weighted average of the ER-negative and the ER-positive centroids, the weights being c and 1-c, where c is the proportion of ER-negative samples in the original dataset (the training data set) used in Parker et al. [20] to define the PAM50 centroids. The samples to be subtyped were then centered by aligning the combined centroid with the centroid of the training dataset. This was achieved by subtracting from the expression vector of each sample the combined centroid and then adding the centroid of the training dataset. Finally, one of the subtype labels luminal A, luminal B, basal, normal-like or HER2 was assigned to each sample by calculating the Spearman correlation between the centered expression vector of the sample and each of the five PAM50 centroids, and selecting the one with the strongest correlation.

### Protein expression and subtypes

Protein levels were determined using reverse phase protein array (RPPA), a platform whereby single protein levels can be measured across a series of samples simultaneously [21]. Altogether 148 primary antibodies were used to detect cancer-related proteins. Frozen tumor samples from patients with sufficient material (from Oslo University Hospital) were lysed by homogenization in lysis buffer containing proteinase inhibitors and phosphatase inhibitors. The tumor lysates were diluted to 1.33 mg/ml concentration as assessed by bicinchonic acid assay and boiled in 1% SDS and 2-mercaptoethanol.

Supernatants were manually diluted in five serial twofold dilutions with lysis buffer. The samples were spotted onto and immobilized on nitrocellulose-coated FAST slides. The slides were probed with 105 primary highly validated antibodies in the appropriate dilution. The signal intensity was captured by a biotin-conjugated secondary antibody and was amplified using the DakoCytomation-catalyzed system (Dako, Glostrup, Denmark). Slides were scanned, analyzed and quantitated using MicroVigene software (VigeneTech Inc., Carlise, MA, USA) to generate spot signal intensities. These were then processed by the R package “SuperCurve” (version 1.01), available at http://bioinformatics.mdanderson.org/OOMPA [22]. The protein concentrations were derived from the supercurve for each sample by curve fitting, log_2_-transformed, and the relative concentrations were normalized by median centering of the samples for each of the antibodies.

RPPA subtypes were obtained using non-negative matrix factorization as done in [9]. Consensus clustering of the samples was performed with an option for four or five groups using Pearson correlation coefficient-based distance and Ward’s minimum variance-based agglomeration method. The best fit on consensus clustering identified five groups: luminal, HER2, basal and reactive I and reactive II, as defined in the TCGA dataset [9]. The RPPA data can be found in Additional file 1a (parts of the data (i.e. total protein antibodies) have been published previously [11]).

### miRNA expression and clusters

miRNA expression was measured using the one-color microarray Human miRNA Microarray Kit (V2) design ID 029297 (Agilent Technologies) according to the protocol supplied by the manufacturer (miRNA Microarray System v2.3). This array contains 887 human miRNAs and is based on miRBase release 14.0. Each array is spotted by 14,907 features (60-mers) including 715 control probes; hence each miRNA is on average replicated approximately 16 times. For labeling and hybridization to the array, 100 ng total RNA was used as input. Scanning was performed on the Agilent Scanner G2565A. Samples were processed using Feature Extraction version 10.7.3.1 (Agilent Technologies). All except two tumors that did not pass array quality control were included in downstream analysis. The data were log_2_-transformed and centered on the 90th percentile using GeneSpring GX v.11.0 (Agilent Technologies). In total, 421 miRNAs were considered to be expressed in the Oslo2 cohort, after filtering out miRNAs detected in fewer than 10% of samples. The miRNA expression data have been submitted to the GEO database [GEO:GSE81000].

In order to identify patient clusters based on miRNA expression, the partitioning algorithm using recursive thresholding (PART) method available in the R package “clusterGenomics” [23] was used. The PART method determines the number of clusters by recursive partitioning of the samples into subgroups. This means that it first attempts to split the data into an optimal number of subgroups by a flat cut of the dendrogram. It then applies the same procedure to each of the clusters identified, to see if any of these can be further split into subgroups. The benefit of this method is that it allows the dendrogram to be split into clusters occurring at different heights in the dendrogram, thus circumventing the limitation of using only flat cuts of the dendrogram to define clusters. The parameter Kmax is a technical parameter defining the maximum number of clusters to be identified at each stage of this procedure. PART was applied with the Pearson correlation coefficient based-distance and complete linkage and parameters Kmax = 4, minSize = 41 and B = 1000.

### Metabolic spectra and clusters

Tumor samples from the Oslo University Hospital of sufficient size to obtain a biopsy sample for high-resolution magic-angle spinning magnetic resonance spectroscopy (HR MAS MRS) were cut to fit into 30-μl inserts containing 3.0 μl of 24.29 mM sodium formate (VWR BDH Prolabo, France) in D_2_O (Armar Chemicals, Switzerland). Each insert was set tightly into a 4 mm optical density (o.d.) MAS zirconium rotor. Samples weighed 7.26 mg on average (2.10–15.60 mg). HR MAS MRS spectra were acquired on a BrukerAvance DRX600 spectrometer equipped with a ^1^H/^13^C MAS probe (Bruker, BioSpin GmbH, Germany). Samples were spun at 5 kHz while kept at a temperature of 5 °C to minimize degradation. A spin-echo one-dimensional experiment with presaturation (cpmgpr1d, Bruker, BioSpin GmbH, Germany) was performed on all samples.

The spectral region between 1.40 and 4.70 parts per million (ppm) containing the major information from low molecular metabolites, excluding lipid-containing regions at 4.36-4.27, 2.88–2.70, 2.30–2.20, 2.09–1.93 and 1.67–1.50, was mean normalized and used for unsupervised hierarchical cluster analysis using the Euclidean distance and Ward’s minimum variance-based agglomeration method (Statistical toolbox, Matlab R2013b, The Mathworks, Inc., USA). The dendrogram was cut to give three metabolic clusters (1–3). Relative intensities from integration of spectral regions were used to measure metabolite levels [6]. The clusters were tested for differences in expression using the Kruskal-Wallis test and corrected for multiple testing with the Benjamini-Hochberg false discovery rate (FDR) [24]. The metabolic data and cluster assignments have been published previously [25].

### Classification of the Oslo2 samples in the ten integrative clusters

Samples in the Oslo2 cohort were assigned to the ten integrative clusters (IntClust) identified in the Molecular Taxonomy of Breast Cancer International Consortium (METABRIC) cohort [3] using integrative clustering (iClustering) [26]. With this aim, the same pipeline as used by Curtis et al. [3] was employed: the pipeline assigns the METABRIC samples in the validation set to the ten clusters obtained from the discovery set. The METABRIC cohort includes 1980 patients (997 in the discovery set, and 983 in the validation set). Integrative clustering is used on the discovery set in order to estimate cluster centroids and the most relevant features (genes) for clustering. METABRIC finally selects ten clusters based on 754 features (39 are segmented copy number aberrations (CNAs) and 715 gene expressions). For assigning the Oslo2 data to these ten clusters, we used the Oslo2 samples for which both CNAs and gene expressions were available (*n* = 291). We also used the same 754 features as in the METABRIC case. As the platforms were different, in order to make the gene expressions and CNAs of the Oslo2 cohort comparable to the METABRIC ones, the Oslo2 data were normalized to have the same mean and standard deviation as the METABRIC data.

In the analysis of Curtis et al. [3] the assignment of the samples in the validation set to the clusters obtained from the discovery set was performed using nearest shrunken centroid (NSC; see [27]), a supervised classification method where cluster centroids are also shrunken. NSC has two phases: in the training phase, ten new shrunken centroids were estimated starting from the original centroids (given in [3]), using the within-cluster standard deviation of each of the 754 features. In the testing phase, each selected Oslo2 sample was assigned to one of the ten shrunken centroids, thus identifying cluster membership.

### Pathway recognition algorithm using data integration on genomic models

The pathway recognition algorithm using data integration on genomic models (PARADIGM) infers distinct biological pathway activity from multiple genomic data (here, mRNA expression and copy number) in a patient sample [5, 28]. The pathway concepts (genes, complexes and abstract processes) are derived from the Pathway Interaction Database [29], BioCarta (http://cgap.nci.nih.gov/Pathways/BioCarta_Pathways), and Reactome databases [30]. Copy number is estimated from AROMA CRMA v2 followed by circular binary segmentation (non-paired CBS) to gene level measurement [31, 32]. The normalized mRNA expression and the 25% most variable copy number data were used for calculating inferred pathway levels (IPL) at five3genomics.com. Clustering was performed by HOPACH 2.10 [33] in R version 3.0.0 [17] and visualized using cluster 3.0 [34] and Java TreeView ver.1.1.6r3 [35]. For the HOPACH hierarchical clustering algorithm, we used correlation distance “cor” and clustering criteria function “med” (median split silhouette) in the mss parameter (mss determines the number of children at each node, to decide what collapsing should be performed at each level, and to determine the main clusters).

### Consensus clustering across the multiple classifications

Consensus Clustering [36] is a method to represent the consensus across multiple classifications. We used the R package “ConsensusClusterPlus” [37] to estimate a final clustering of the Oslo2 cohort starting from multiple classifications. Only the samples that were clustered with at least two methods were included in the consensus clustering analysis. Consensus clustering was run using hierarchical clustering with normalized Manhattan distance and Ward linkage, and the final choice resulted in six clusters. The number of COCA clusters was selected by inspecting the average silhouette value [38] associated to the final grouping, which showed a global maximum when selecting six consensus clusters. The clusters selection criterion suggested by the authors proposing the COCA method [36] was also examined to support this choice.

### Association of COCA clusters to clinical parameters and correlation to molecular subtype levels

To associate the identified COCA clusters to clinical parameters, a Chi-squared association test was used. To assess the correlation between COCA clusters and molecular subtype levels the Pearson correlation coefficient was calculated. This was done by first coding each sample 0/1 if it belonged (or not) to each molecular subtype and each COCA cluster and then correlating a given 0/1 COCA cluster vector with a given 0/1 molecular subtype vector.

Additional file 2a contains further methodological description. All computational analyses were performed in R [17] unless otherwise specified.

## Results

### Samples and clinical data

This collection of 425 primary breast tumors represents a consecutive breast cancer cohort. For each molecular level, different numbers of patients were classified (in descending order): miRNA expression (*n* = 423), PAM50 gene expression (*n* = 377), complex arm aberration index (CAAI) (*n* = 349), PARADIGM (*n* = 312), IntClust (*n* = 291), metabolic profiling (*n* = 233) and RPPA (*n* = 173). Altogether 80 patients had available data from all seven levels. The various molecular levels are further described subsequently. An overview of the clinicopathological data on the patients is provided in Additional file 2b and the tumor sample classifications are listed in Additional file 1b.

### PAM50 gene expression subtypes

**Fig. 1:**
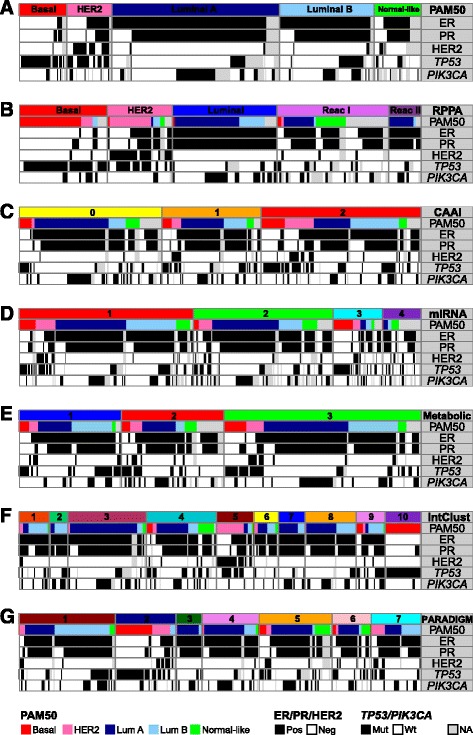
The seven input levels for integrative clustering and association with clinical/molecular classifications. Original subtypes/clusters of each input level and corresponding PAM50 gene expression subtype, estrogen receptor (*ER*), progesterone receptor (*PR*) and epidermal growth factor receptor 2 (*HER2*) status, and *TP53* and *PIK3CA* mutation status of the tumor samples. The tumor samples are sorted in the following order: molecular level, PAM50 gene expression subtype, ER, PR, HER2, *TP53* and *PIK3CA* status. **a** PAM50 gene expression subtypes (n = 377). **b** Reverse-phase protein array (*RPPA*) subtypes (n = 173). **c** Complex arm aberration index (*CAAI*) subtypes (n = 349). *0* no CAAI events, *1* one CAAI event, *2* at least two CAAI events. **d** miRNA clusters (n = 423). **e** Metabolic clusters (n = 233). **f** Integrated clusters (*IntClust*; n = 291). **g** Pathway recognition algorithm using data integration on genomic models (*PARADIGM*) clusters (n = 312). *Lum* luminal, *Pos* positive, *Neg* negative, *Mut* mutant, *Wt* wild-type, *NA* not applicable

Tumor samples with mRNA expression data available were classified into gene expression-based subtypes based on the PAM50 model [20] (Fig. 1a). PAM50 subtyping classifies breast tumors into one of five subtypes on the basis of the measured expression of 50 selected genes. It compares the tumor gene-expression vector with five centroids that represent the subtypes. The nearest centroid is identified, and the corresponding subtype label is assigned to the tumor. Among the tumors with available mRNA expression data the majority of samples were classified as luminal samples, comprising 41.6% luminal A and 23.6% luminal B samples. The remaining samples were classified as basal (12.0%), HER2-enriched (11.1%) and normal-like (11.7%).

### Classification based on protein expression

The RPPA subtypes are based on clustering the expression levels of 148 selected cancer-associated proteins (Additional file 1a). The five RPPA subtypes found in the Oslo2 dataset have been previously defined [9]. There was strong concordance between three of these - basal, HER2 and in part, the luminal subtype - and the corresponding gene expression-based PAM50 subtypes (Fig. 1b). The remaining two subtypes, reactive I and reactive II, originally had protein expression profiles characteristic of the microenvironment and/or cancer-activated fibroblast [9] and have recently been shown to represent a high stromal content in the context of a highly differentiated tumor [39]. Here, they overlapped mostly with the luminal A and normal-like PAM50 subtypes.

### CAAI scoring from CNA data

The CAAI index identifies complex architectural alterations on chromosome arms, which are characterized by physically tight clusters of breakpoints with large changes in amplitude [18]. Identification of CAAI events is a measure of the local distortion on chromosome arms and has previously been shown to be an independent prognostic marker [18, 40]. Among the 349 tumors with available CAAI scores, 35.5% had no CAAI event (CAAI <0.5 for all arms), 24.7% had one CAAI event (one chromosome arm with CAAI ≥ 0.5), and 39.8% had at least two CAAI events (Fig. 1c). The three CAAI groups all have representatives from each of the PAM50 subtypes. As expected, HER2-positive tumors were classified as CAAI 1 or CAAI 2 as they often have amplification of the 17q region harboring the *ERBB2* gene [41].

### miRNA expression classification

By applying the PART algorithm [23] to separate the tumor samples into clusters based on miRNA expression, we identified four clusters (Fig. 1d and Additional file 3). Tumors of the luminal expression subtypes (luminal A and B) were mostly found in cluster 1 and 2, while basal-like tumors were mainly found in cluster 3, which was dominated by ER-/PR-negative tumors. HER2-positive tumors were not clustered together; rather they were distributed across the clusters 1, 2 and 3 (Additional file 3). miRNA cluster 4 was a mixture of mostly normal-like and luminal samples; however, a subset of tumors in this cluster did not have PAM50 expression subtype classification due to lack of available mRNA expression data. miRNAs that were differentially expressed between the clusters are listed in Additional file 4.

### Classification of tumors based on metabolic profiles

HR MAS MRS was performed on 233 breast tumors in order to extract tumor metabolic profiles [6]. Unsupervised hierarchical clustering analysis separated the tumors into three metabolic clusters (Fig. 1e; [6]). The clusters differed in the expression of metabolites involved in phospholipid metabolism, glycolytic activity and glutaminolysis. Cluster 1 tumors had the highest levels of the choline-containing metabolites glycerophosphocholine (GPC) and phosphocholine (PCho). Altered choline metabolism is an emerging hallmark of malignant transformation [42] and both GPC and PCho have previously been confirmed to be elevated in tumor tissue compared to non-involved breast tumor tissue [43]. Cluster 1 tumors also had evidence of increased glycolytic activity, with low levels of glucose and high levels of lactate approaching statistical significance when compared to cluster 2 tumors. However, cluster 3 tumors had a more apparent glycolytic switch, with the highest levels of lactate among all three clusters, combined with high alanine and low glucose levels. Cluster 2 tumors expressed significantly higher levels of glucose and lower levels of lactate and alanine, indicative of lower glucose consumption and glycolytic activity. Increased glucose consumption has been shown to correlate with poor prognosis and tumor aggressiveness [44], inferring that patients with cluster 2 tumors have a better prognosis than patients with cluster 1 or cluster 3 tumors. There was no correlation between the three metabolic clusters and PAM50 subtypes.

### Integrated clusters

iClustering [26] is a sparse clustering method originally used to perform breast tumor subtyping in the METABRIC cohort [3]. The subtyping is based on 754 selected features (715 gene expression and 39 copy number values). Among the samples in the Oslo2 cohort, data from 291 tumors were selected according to the availability of gene expression and copy number data, and they were assigned to the 10 IntClusts identified in the METABRIC cohort. The IntClust assignments and clinical annotations of these samples are shown in Fig. 1f. The assignment to IntClust centroids resulted in 22 samples being assigned to IntClust 1, 13 to IntClust 2, 57 to IntClust 3, 51 to IntClust 4, 27 to IntClust 5, 18 to IntClust 6, 19 to IntClust 7, 37 to IntClust 8, 21 to IntClust 9 and 26 to IntClust 10. The distribution into IntClusts was similar to that in the METABRIC cohort. Some of the IntClusts correlated with the PAM50 subtypes, for example IntClust 3 and 5 were mainly composed of luminal A and HER2-enriched tumors, respectively, while IntClust 10 consisted entirely of basal-like tumors.

### Patient clusters based on pathway activity levels

The PARADIGM algorithm infers patient-specific pathway activity by incorporating gene expression and copy number data with pathway information [28]. Clustering the inferred pathway activities resulted in identification of seven patient clusters of varying size (Fig. 1g). IPLs for the top deregulated pathway entities across the clusters are visualized as a heatmap in Additional file 5. The pathways with levels that most strongly contributed to these groups were those related to transcription factors such as ER, E2F1, Myb, Myc/Max, Jun/Fos and TP53. A list of the top 500 pathway entities defining the seven clusters is supplied in Additional file 6.

### Multilevel classification using consensus clustering

**Fig. 2:**
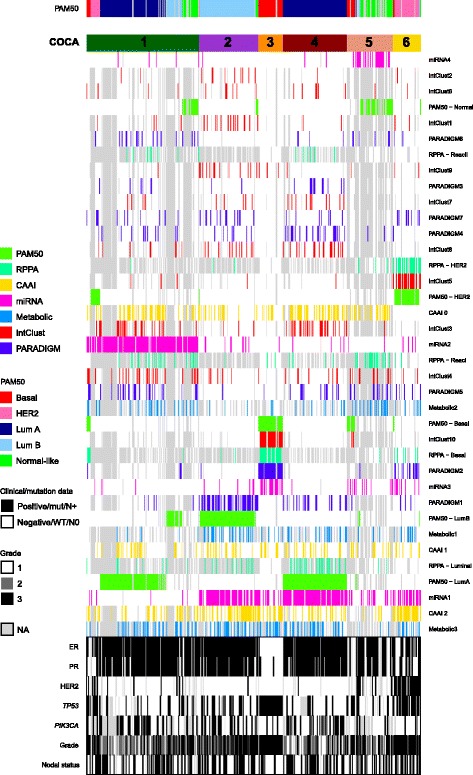
Cluster-of-clusters analysis (*COCA*) identifies six major groups based on seven molecular input levels. Consensus clustering was used to cluster 419 primary breast cancers in the Oslo2 study. The six resulting COCA clusters are numbered and the corresponding PAM50 subtype indicated (*top*). Heatmap representation of the subtypes/clusters independently defined: PAM50 mRNA subtypes, reverse-phase protein array (*RPPA*) expression subtypes, complex arm aberration index (*CAAI*) subtypes based on copy numbers, miRNA clusters, metabolic clusters, pathway recognition algorithm using data integration on genomic models (*PARADIGM*) clusters and integrated clusters (*IntClust*). *Colored bar* indicates membership of a subtype/cluster type, *white* indicates no membership to a given subtype and *gray* represents data not available (*NA*). The *rows* in the heatmap are ordered according to clustering. Clinical annotation of the tumors is shown (*bottom*). *HER2* human epidermal growth factor receptor 2, *Lum* luminal, *Mut* mutant, *WT* wild-type

COCA is a clustering method whereby cluster assignments found on multiple data levels are jointly used for subtype classification. It was first applied on TCGA breast cancer data [9] and then later on a TCGA pan-cancer study [10]. The purpose of using this method is to explore the higher-order composition of tumors, which might not be visible when considering one data level at a time, and to see how multiple molecular levels are associated when integrated. In this study, single-level classifications based on gene/protein/miRNA expression, CNAs and metabolic profiles were clustered together with classifications based on the combined analysis of copy number and gene expression using iClustering and PARADIGM. Unsupervised consensus clustering of seven levels of classifications of the Oslo2 data (*n* = 419 tumors with at least two data levels available) revealed six clusters of varying size (Fig. 2). The tumor sample classifications to COCA clusters are listed in Additional file 1b.

In order to investigate the association between the various molecular subtype levels and COCA clusters, the Pearson correlation coefficient between each molecular subtype level and each COCA cluster was calculated. This was done by coding each sample 0/1 if it belonged (or not) to each molecular subtype and each COCA cluster (Table 1 and Additional file 1c). Interestingly, miRNA clusters and PAM50 subtypes were the levels that were most frequently strongly correlated with the COCA clusters (Table 1). For the COCA cluster 3 (basal) and COCA cluster 6 (HER2) there was strong correlation to more levels than for the other clusters. This suggests that the luminal clusters can be further subdivided by the COCA clustering approach using data from multiple platforms.

**Table 1:** Top five molecular subtype levels ranked according to correlation to each COCA cluster

COCA cluster 1	COCA cluster 2	COCA cluster 3	COCA cluster 4	COCA cluster 5	COCA cluster 6
miRNA2 (**0.80**)	PAM50 - LumB (**0.82**)	IntClust10 (**0.83**)	PAM50 - LumA (**0.63**)	miRNA4 (**0.55**)	PAM50 - HER2 (**0.73**)
PAM50 - LumA (0.23)	PARADIGM1 (**0.55**)	PAM50 - Basal (**0.76**)	miRNA1 (**0.45**)	PAM50 - Normal (**0.43**)	IntClust5 (**0.69**)
PARADIGM6 (0.15)	miRNA1 (**0.32**)	PARADIGM2 (**0.71**)	RPPA - Luminal (**0.30**)	RPPA - ReacI (0.25)	RPPA - HER2 (**0.68**)
IntClust4 (0.12)	IntClust1 (0.28)	RPPA - Basal (**0.67**)	IntClust3 (0.29)	Metabolic2 (0.15)	CAAI3 (0.28)
PARADIGM5 (0.12)	IntClust9 (0.27)	miRNA3 (**0.40**)	PARADIGM4 (0.27)	miRNA3 (0.13)	PARADIGM2 (0.28)

Values in parentheses are the Pearson correlation values. Correlation ≥0.3 (p < 0.001 for all) is indicated in bold font. *COCA* cluster-of-clusters analysis, *LumA* luminal A, *PARADIGM* pathway recognition algorithm using data integration on genomic model, *IntClust* integrated clusters, *RPPA* reverse-phase protein array, *CAAI* complex arm aberration index

#### COCA cluster 1

COCA cluster 1 was the largest in size (*n* = 141) and tumors of cluster 1 were most strongly correlated with miRNA cluster 2 (*r* = 0.80), with 125 (88.7%) of the tumors in cluster 1 assigned to this miRNA cluster. Interestingly, as more than half of the tumors in cluster 1 were classified as luminal A samples (*n* = 75; 53.2%) and all other luminal A samples (*n* = 82) except two were found in cluster 4, this revealed a split in the luminal A tumors. Although this cluster was dominated by luminal A tumors, cluster 1 was the most mixed cluster according to mRNA expression subtype with representatives from all subtypes (Additional file 1d). Using the chi-squared test to assess the association between the six COCA clusters and clinical parameters, cluster 1 was associated with grade (*p* = 0.001; mostly grade 2 and 3), ER (*p* = 0.009; mostly ER-positive), PR (*p* = 0.042; mostly PR-negative) and *TP53* status (*p* = 0.018; mostly wild-type) (Additional files 1e and 7).

#### COCA cluster 2

COCA cluster 2 was most strongly correlated to the luminal B subtype (*r* = 0.82; corresponding to 93.2% of the tumors in the cluster), PARADIGM 1 cluster (*r* = 0.55; high activation of the ER-alpha network and targets of C-MYC transcriptional activation, low activation of JUN/FOS signaling and targets of C-MYC transcriptional repression) and miRNA 1 cluster (*r* = 0.32) (Table 1). Furthermore, cluster 2 was significantly associated with grade (*p* = 0.018; grade 2 and 3), HER2 (*p* = 0.023; almost exclusively HER2-negative), ER (*p* < 0.001; all positive) and PR status (*p* = 0.025; mostly positive) (Additional files 1e and 7).

#### COCA cluster 3

Overall, tumors in COCA cluster 3, which represented the basal subgroup (93.5% of the tumors; correlation with the basal-like subtype = 0.76) were the most strongly correlated. COCA cluster 3 was most strongly correlated to the IntClust 10 group (*r* = 0.83), which has previously been associated with younger age at diagnosis, high-grade and large tumors [3]. The tumors in cluster 3 were also strongly correlated to the PARADIGM 2 cluster (*r* = 0.71), the basal RPPA subtype (*r* = 0.67) and the miRNA 3 cluster (*r* = 0.40) (Table 1). Furthermore, cluster 3 was associated with grade (*p* < 0.001; all except one tumor were of the highest grade), ER (*p* < 0.001; mostly negative), PR (*p* < 0.001; mostly negative) and *TP53* status (*p* < 0.001; all except for two tumors were mutated) (Additional files 1e and 7). All tumors in cluster 3 were HER2-negative and this cluster also represented the largest proportion of patients diagnosed at a younger age (<50 years) (Additional file 7).

#### COCA cluster 4

COCA cluster 4 exclusively comprised tumors of the luminal A expression subtype, and correspondingly this level was the most strongly correlated (*r* = 0.63). Cluster 4 tumors were also correlated with miRNA cluster 1 (*r* = 0.45) and with the RPPA luminal group (*r* = 0.30; Table 1). Furthermore, this cluster was significantly associated with grade (*p* < 0.001; mostly grade 1 and 2), ER (*p* < 0.001; all positive), PR (*p* = 0.003; mostly positive), HER2 (*p* = 0.007; all except one were negative) and *TP53* status (*p* = 0.001; mostly wild-type) (Additional files 1e and 7). With only 12.7% *TP53* mutated tumors, this was the cluster with the lowest frequency of *TP53* mutations.

#### COCA cluster 5

COCA cluster 5 was most strongly correlated with miRNA cluster 4 (*r* = 0.55), and the normal-like subtype (*r* = 0.43; 43.1% of the tumors). Tumors of the basal-like and luminal subtypes were also present, but notably, this cluster contained a substantial subgroup of tumors (34.5%) that were lacking PAM50 subtype classification (and thus also IntClust and PARADIGM classifications). Cluster 5 was significantly associated with histology (*p* = 0.017; Additional file 1e); this cluster was the most histologically diverse cluster with the lowest frequency of ductal carcinomas (62.7%) compared to the other clusters and with the highest frequency of lobular (17.6%) and ductal carcinoma *in situ* (DCIS) samples (11.8%) (Additional file 7).

#### COCA cluster 6

COCA cluster 6 was the smallest cluster in size, with only 35 tumors. These were mainly of the HER2-enriched subtype (*r* = 0.73; 82.9% of the tumors), but also including a few tumors from the basal-like, luminal B and normal-like subtypes (Additional file 1d). Cluster 6 was also highly correlated with the IntClust 5 group (*r* = 0.69) which correspondingly represented tumors with amplification of the *ERBB2* gene in the original METABRIC cohort [3], and to the RPPA HER2 subtype (*r* = 0.68). Furthermore, this cluster was associated with grade (*p* < 0.001; mostly grade 3), ER, PR, HER2 and *TP53* (all *p* < 0.001) and *PIK3CA* status (*p* = 0.049) (Additional files 1e and 7). Tumors in this cluster were mainly high grade (84.6% grade 3), and this cluster had the second highest frequency of *TP53* mutations (69.2%) after the basal-like cluster 3. In contrast, it was the cluster with the lowest frequency of *PIK3CA* mutations (8.0%).

### Contribution from the different molecular levels in varying degrees

**Fig. 3:**
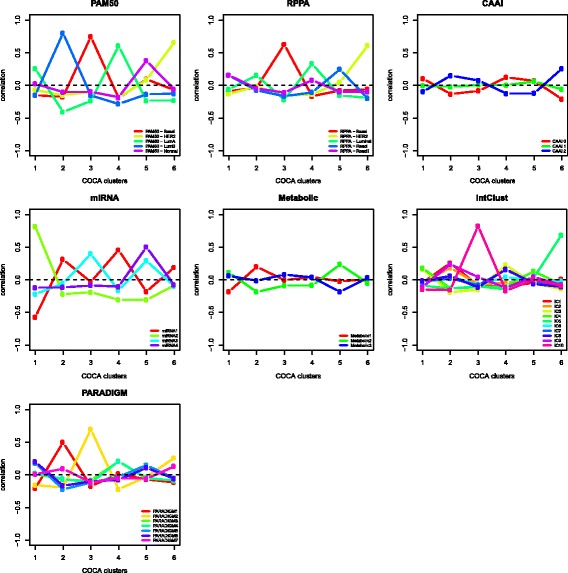
Correlation between cluster-of-clusters analysis (*COCA*) clusters and molecular input levels. Pearson correlation coefficient (*y-axis*) calculated between each molecular subtype level and each COCA cluster (*x-axis*) by coding membership to a cluster as 1 and 0 otherwise. Each *panel* represents one molecular input level to the COCA analysis. *RPPA* reverse-phase protein array, *CAAI* complex arm aberration index, *PARADIGM* pathway recognition algorithm using data integration on genomic models, *IntClust* integrated clusters, *HER2* human epidermal growth factor receptor 2, *Lum* luminal

Figure 3 summarizes the correlation values between all molecular levels and all six COCA clusters, showing the contribution from each level. All the five PAM50 subtypes and the four miRNA clusters had maximum correlations that were above 0.3. Three of the five RPPA subtypes (basal, HER2 and luminal) also showed high maximums, and the same was true for two out of seven PARADIGM clusters (cluster 1 and 2) and two out of ten IntClusts (IntClusts 5 and 10). Neither of the three CAAI groups, nor the three metabolic clusters showed high correlations to any COCA cluster.

### miRNA expression split the luminal A tumors

Interestingly, the luminal A tumors were split into COCA cluster 1 and COCA cluster 4. This was most evidently due to different miRNA cluster assignment (Fig. 2). Therefore, miRNA differential expression was calculated between the luminal A tumors in COCA cluster 1 versus COCA cluster 4. Altogether, 71 miRNAs were identified as differentially expressed (Additional file 1f). To study the functional role of these miRNAs that were differentially expressed between luminal A tumors in COCA cluster 1 and COCA cluster 4, we performed miRNA gain-of function studies in the ER-positive cell line MCF-7. Proliferation, apoptosis, viability, phosphorylated AKT (p-AKT) levels and ER levels were used as endpoints (Additional file 8).

Of the 71 miRNAs assessed, 13 miRNAs had functional effects when overexpressed in MCF-7: miR-23a*, miR-33b, miR-33b*, miR-125a-3p, miR-452, miR-492, miR-494, miR-526b, miR-582-5p, miR-654-5p, miR-765, miR-934 and miR-1226*. Of these, mir-33b and miR-582-5p were higher expressed in COCA cluster 4, while the rest were higher expressed in COCA cluster 1. Interestingly, miR-1226* had several tumor-suppressor features: overexpression led to reduced cell viability and proliferation and increased apoptosis. Furthermore, overexpression of miR-1226* led to reduced ER and p-AKT. Overexpression of miR-452 and miR-526b led to both reduced proliferation and p-AKT.

In order to further couple the 71 differentially expressed miRNAs to biological function, correlation between these and the mRNA expression of all genes was calculated. Retaining the genes with the highest absolute correlation (Spearman rank correlation > |0.4|) resulted in a list of 1808 unique genes (Additional file 1g). Further, we tested which of these 1808 genes were significantly differentially expressed between luminal A tumors in COCA cluster 1 versus COCA cluster 4 and identified 1323 genes (Benjamini-Hochberg corrected *p*-value <0.05), of which 473 genes were upregulated in luminal A tumors in COCA cluster 1 (compared to luminal A tumors in COCA cluster 4) and 850 genes were upregulated in COCA cluster 4 (compared to luminal A tumors in COCA cluster 1; Additional file 1g). Finally, IPA was used to test for enrichment of pathways within the two lists of genes upregulated in luminal A tumors in the respective COCA clusters. After correcting for multiple testing, no pathways were enriched among the genes upregulated in luminal A tumors in COCA cluster 1. In the luminal A tumors in COCA cluster 4, 15 pathways were enriched among the upregulated genes (Benjamini-Hochberg-corrected *p*-value <0.05 (Fisher’s exact test); Additional file 9). The top five most significantly enriched pathways included mitochondrial dysfunction, EIF2 signaling, oxidative phosphorylation, protein ubiquitination pathway, and androgen signaling (Additional file 1h).

Further, using chi-squared tests to assess if the luminal A samples in the two clusters were also different with respect to the other COCA input levels, both PARADIGM clusters and RPPA subtype distributions were statistically significant (*p*-value <0.001 and *p*-value = 0.002, respectively). The largest proportion of luminal A samples in COCA cluster 1 (36%) belonged to the PARADIGM 4 cluster, while the largest in COCA cluster 4 belonged to the PARADIGM 3 cluster (29%). The most striking difference in pathway activity levels between these two PARADIGM clusters was the lower activation of JUN/FOS-associated pathways in PARADIGM 3 (luminal A tumors in COCA cluster 4) and higher activation in PARADIGM 4 (luminal A tumors in COCA cluster 1; Additional file 5).

Although all tumors were classified as luminal A based on mRNA expression, the RPPA subtype distribution was different; among the tumors with RPPA classification most of the COCA cluster 1 tumors were classified as reactive I (47%) and reactive II (26%), while most of the COCA cluster 4 tumors were classified as luminal (71%) (note, only 34% of the luminal A tumors in COCA clusters 1 and 4 were assigned an RPPA subtype).

To further investigate the RPPA subtype-defined differences between the luminal A samples, *t* tests were used to assess the difference in protein expression. Of 148 proteins (antibodies) tested, 6 were statistically significant (Benjamini-Hochberg adjusted *p*-value <0.05): cleaved Caspase 9, 53BP1, AMPK-alpha, GATA3, Rad51, and p90RSK (phosphorylated at T359 and S363) (Additional file 10).

To assess potential interactions between these six proteins and the 71 differentially expressed miRNAs between luminal A tumors in COCA cluster 1 and 4, a list of *in silico* predicted target genes of the 71 miRNAs were obtained and overlaid with the 6 proteins. Five of the six proteins were predicted to be targets of at least one of the differentially expressed miRNAs resulting in a list of ten potential miRNA-protein interactions (Additional file 1i). Correlation analysis between miRNA and protein expression showed that of the ten potential interactions, the presence of both positive and negative correlation suggests the potential for both inhibitory and stimulating relationships between these miRNAs and proteins.

There was no statistically significant difference in clinicopathological parameters between luminal A tumors in COCA cluster 1 compared to COCA cluster 4 (*p*-value >0.05, chi-squared association tests). Furthermore, there was no statistically significant difference in tumor percentage, or in correlation with the PAM50 luminal A centroid or with the next nearest subtype (data not shown).

### Prognostic differences between luminal A tumors

As the Oslo2 cohort has been established relatively recently, extensive follow-up data are not yet available. To investigate the prognostic potential of the 71 miRNAs distinguishing the two groups of luminal A tumors, luminal A tumors from four other datasets with available miRNA expression and long-term follow up were assessed; METABRIC (*n* = 447), TCGA (*n* = 230), the Danish Breast Cancer Cooperative Group (DBCG) (*n* = 33) and the Oslo Micrometastasis cohort (Micma) (*n* = 29). Of the 71 miRNAs in the signature, 68 miRNAs were available in the METABRIC cohort and 56 miRNAs were available in the three latter datasets.

**Fig. 4:**
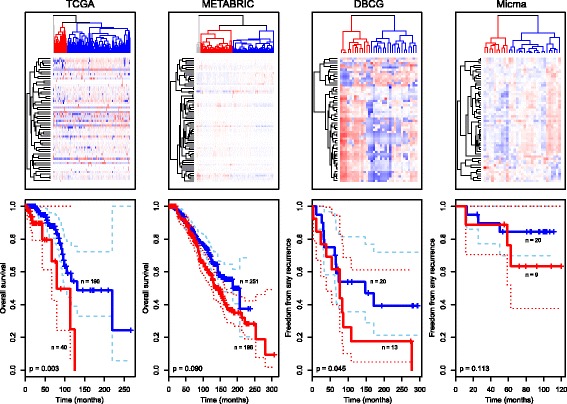
miRNA expression separates luminal A tumors into clusters with different outcomes. *Top panel* Luminal A tumors in The Cancer Genome Atlas (*TCGA*), Molecular Taxonomy of Breast Cancer International Consortium (*METABRIC*), Danish Breast Cancer Cooperative Group (*DBCG*) and the Oslo Micrometastasis cohort (*Micma*) breast cancer cohorts were clustered based on the expression of selected miRNAs using Pearson correlation and complete linkage (patients in *columns* and miRNAs in *rows*). *Bottom panel* Kaplan-Meier survival curves for the *red* and *blue* clusters in the *top panel*. The *p*-values are from log-rank tests (METABRIC *p*-value was adjusted for hospital site and DBCG *p*-value was adjusted for radiation therapy and lymph node status). *Dashed lines* indicate confidence intervals for the survival curves

Clustering the luminal A tumors in each of the datasets on the expression of these miRNAs revealed two main clusters in each cohort (Fig. 4). A log-rank test was used to assess if the survival curves were different in the two groups. Indeed, in TCGA and the DBCG the split in the luminal A tumors based on the expression of the 56 miRNAs was related to differences in outcome (overall survival and freedom from any recurrence, log-rank *p*-values 0.003 and 0.045, respectively: the DBCG log-rank *p*-value was adjusted for radiation therapy and lymph node status and the adjusted hazard ratio was 2.52 (95% CI 1.02–6.24)). In the METABRIC cohort there was a trend towards differences in prognosis between the two clusters for overall survival, but the log-rank test was not significant after adjusting for hospital (*p* = 0.090 after stratification). There was no prognostic difference in the Micma cohort, possibly due to the small sample size (*p*-value = 0.113).

RPPA subtypes of the TCGA tumors [9] confirmed the findings from Oslo2 that the constitution of the luminal A tumors split according to miRNA were different with respect to protein-defined subtypes; TCGA tumors in the cluster with worse prognosis represented 43% luminal tumors (scored as luminal A or luminal A/B RPPA subtype), 3% reactive I tumors and 5% reactive II tumors (Additional file 1j). On the other hand, TCGA tumors in the cluster with a better prognosis represented 22% luminal tumors, 12% reactive I tumors and 3% reactive II tumors. Thus, the cluster with a better prognosis represented a larger proportion of reactive tumors and fewer luminal tumors compared to the cluster with a poorer prognosis. According to these findings the luminal A tumors in COCA cluster 1 may have a better prognosis than those in COCA cluster 4. Furthermore, of the six proteins that were differentially expressed between luminal A tumors in Oslo2 (Additional file 10), four were present in the TCGA data. Three of these, GATA3, RPS6KA1 and PRKAA1 followed the same trend with respect to different expression (high/low) in TCGA, but only RPS6KA1 was statistically significantly differentially expressed (*t* test *p*-value = 0.01; GATA3 *p*-value = 0.08 and PRKAA1 *p*-value = 0.14).

## Discussion

The input to the COCA analysis was seven different classifications of breast tumors; the PAM50 subtype, RPPA subtype, metabolic cluster, miRNA cluster, CAAI, PARADIGM and IntClust. The five former were single-molecular-level classifications, while the IntClust and PARADIGM classifications were based on the combined analysis of copy number and expression data in two different ways; iClustering assigned each tumor to one of ten IntClusts derived from the METABRIC cohort, while PARADIGM identified patient clusters based on inferred pathway activity levels. The distance in the consensus clustering method was normalized so that the different layers would be comparable in terms of number of missing values associated with each layer. Furthermore, a strength of the current work is the processing of the tumors where cutting and blending the tissue before dividing it into DNA, RNA and protein isolation ensured representative and comparable molecular data.

We identified six COCA clusters in our analysis. Considering the ranking of the molecular levels based on correlation with the COCA clusters (Table 1), PAM50 subtypes and miRNA clusters were the most strongly correlated; all of the five subtypes and four miRNA clusters were present among the strongest correlations. The PAM50 subtypes have been recognized as a robust classifier [9]. miRNA expression has previously been associated with both gene expression-based subtypes and with clinical parameters [45, 46], but subtypes have not yet been “formally” established based on miRNA expression. Interestingly, on ranking all the molecular levels, COCA clusters 1 and 5 were most strongly correlated with miRNA clusters 2 and 4, respectively, suggesting an important role for miRNAs in the separation of breast tumors.

In the TCGA breast study [9], seven miRNA expression-defined subtypes were identified by consensus non-negative matrix factorization clustering. Except for two of the clusters, each of the clusters was a mixture of the PAM50-defined subtypes. As the four identified consensus clusters mainly recapitulated the PAM50 subtypes, the miRNA clusters were not given a dominant role in the TCGA study. Importantly, this particular study contained very few normal-like samples (1%), and thus the tumor distribution was different from the Oslo2 cohort consisting of 11% normal-like tumors. Similarly as in the TCGA study, the basal-like COCA cluster 3 had the most distinct signature with the strongest associations with several levels; IntClust 10, PAM50 basal subtype, PARADIGM cluster 2, RPPA basal subtype, and miRNA cluster 3 were all strongly correlated with this cluster.

There was also correlation between the COCA clusters and some of the RPPA subtypes, PARADIGM clusters and IntClusts; however, neither the metabolic clusters nor the CAAI subtypes were strongly correlated with any of the COCA clusters, suggesting that grouping based on metabolic clusters and complexity of DNA rearrangements are less strongly associated with the molecular subtypes driven by the other platforms.

Luminal A tumors represent the most frequent breast cancer subtype (approximately 40% of all cases). Although considered to have the best prognosis, the luminal A subtype is also characterized as the most heterogeneous group, both clinically and molecularly [9, 47]. Some patients with this disease subtype suffer from relapse and may benefit from adjuvant treatment, while others risk unnecessary over-treatment with adverse side effects. Furthermore, survival curves for patients with luminal A tumors suggest that the risk of delayed local relapse and/or distant metastasis persists over long time periods compared to other subtypes [48].

Heterogeneity at the molecular level was found for luminal A tumors in terms of mRNA expression, mutation spectrum and copy number changes in the TCGA breast cancer study [9]. In the METABRIC study, which identified ten integrative clusters across breast cancers, luminal A tumors were separated mainly into three distinct subgroups which were found to be driven by specific genomic aberrations [3]. Ciriello et al. [47] analyzed copy number and mutation profiles in luminal A tumors and identified four major subtypes with distinct alterations and clinical outcomes.

Being able to distinguish subgroups of luminal A tumors is an important task and may potentially improve the choice of therapeutic approaches and prediction of clinical outcomes. In this respect, the split of the Oslo2 luminal A samples into COCA clusters 1 and 4 may suggest a novel refinement of this group. It was interesting to see that the two luminal A clusters were associated with different miRNA clusters and that overexpression of 13 of the 71 differentially expressed miRNAs in the luminal cell line MCF-7 directly showed functional effects that are important for cancer cell survival. The putative tumor-suppressor miRNA miR-1226*, which was more highly expressed in COCA cluster 1 and for which overexpression resulted in both decreased proliferation, cell viability, ER and p-AKT levels, and increased apoptosis, has previously been found to target and downregulate expression of the MUC1 oncoprotein and induce cell death [49].

Although long-term follow up of the Oslo2 patients is not yet available, it was intriguing to see that in patients from four other cohorts, luminal A tumors formed two separate clusters when clustered on the same miRNAs differentially expressed in Oslo2. Furthermore, there was a prognostic difference between the patient clusters in the TCGA and DBCG cohorts. From the other molecular differences identified between those clusters, it may seem that the tumors in COCA cluster 4 are more “core” luminal, as they were more frequently assigned to the luminal protein-based subtype and with higher protein expression of the luminal marker GATA3 [50].

The majority of the luminal A tumors belonging to COCA cluster 1 were of the RPPA-defined reactive I and II subgroups, which were characterized as being highly differentiated tumors with high expression of stromal proteins due to high numbers of stromal cells, lower levels of GATA3 protein compared to other tumors classified as luminal A/B from gene expression and with a favorable clinical outcome [39]. This difference in association between the RPPA subtype and the luminal A clusters separated by miRNA expression was also seen in the TCGA cohort in which RPPA subtypes were available. Coupling this to outcome data, it seems that the cluster with more tumors classified as the reactive subtype is associated with a better prognosis. The 71 miRNA signatures would need further development to serve as a diagnostic test for patients with luminal A tumors. miRNA-based tests may be beneficial as miRNA molecules are short and relatively stable [51] and can be successfully applied on, for example, formalin-fixed paraffin-embedded tissue. Further studies for better understanding of the underlying biology and the possible role of miRNAs as markers to separate luminal tumors with different clinical outcome or response to therapy is needed and will be exciting to follow up.

## Conclusions

In summary, the six integrated subtypes identified in the current study underline the heterogeneity of breast cancer, but also show that finer subdivisions of subtypes might not be a second-order effect, but might be as strong as the established taxonomies. We were able to validate the split of the luminal A tumors found in Oslo2 based on miRNA expression in four other cohorts and in two of them, TCGA and DBCG, the resulting clusters showed differences in disease outcome. Increasing the knowledge of the heterogeneity of the luminal A subtype of breast cancer revealing more detailed subcategorizations may add to informing therapeutic choices, evidently bringing improved treatment for this largest subgroup of breast cancer.

## Additional files

Additional file 1: **a** Log_2_-transformed and median-centered RPPA data. **b** Molecular classifications of the 425 Oslo2 tumors. **c** Pearson correlation values and corresponding *p*-values calculated for correlation between each molecular subtype level and each COCA cluster by giving 0/1 numerical values to the binary categorical variables. **d** PAM50 distribution in total number and percentage in each of the six COCA clusters. **e** *P*-values for association between each of the six COCA clusters and clinical/molecular classification of the tumors (chi-squared association test). The *p*-values are Bonferroni-corrected for multiple testing. **f** miRNAs significantly differentially expressed (Benjamini-Hochberg adjusted *p*-value <0.01 and log_2_ |fold-change| >1) between luminal A samples in COCA cluster 1 and COCA cluster 4. **g** Annotation of the 1808 genes that were correlated with the 71 miRNAs differentially expressed between luminal A tumors in COCA cluster 1 versus COCA cluster 4 (absolute Spearman correlation >0.4). **h** Pathways enriched among the 850 genes upregulated in luminal A tumors in COCA cluster 4. **i** miRNAs differentially expressed between luminal A tumors in COCA cluster 1 vs COCA cluster 4 and predicted target genes that were among the proteins differentially expressed between luminal A tumors in COCA cluster 1 vs COCA cluster 4. **﻿j﻿** Comparison of the composition of RPPA-defined subtypes in luminal A tumors separated on miRNA expression. The RPPA subtype data are taken from [9].(XLSX 871 kb)

Additional file 2: **a** Supplementary methods. **b** Summary of clinicopathological properties of the 425 primary breast tumors in the Oslo2 cohort. (PDF 137 kb)

Additional file 3: Four miRNA patient clusters (1‒4) derived from clustering the expression of 421 miRNAs using Pearson correlation and complete linkage. The PART algorithm was used to identify clusters [23]. (PDF 136 kb)

Additional file 4: *P*-values and log_2_ fold-change resulting from testing miRNA differential expression between one miRNA cluster versus all other clusters using Wilcoxon rank-sum tests. *P*-values are corrected for multiple testing using Benjamini-Hochberg false discovery rate correction. (XLSX 85 kb)

Additional file 5: Oslo2 tumors sorted according to membership of each of seven PARADIGM clusters (columns) with heatmap representation of the top 253 most deregulated pathway entities (IPLs) across the clusters (*rows*; filtering out IPLs with activity -0.25 > x < 0.25). IPL name details can be seen by zooming in. (PDF 2274 kb)

Additional file 6: *P*-values and statistics from analysis of variance identifying the top 500 pathway entities defining the seven PARADIGM clusters. (XLSX 39 kb)

Additional file 7: Clinical and molecular distribution in the six COCA clusters. (PDF 7 kb)

Additional file 8: Functional studies of miRNAs differentially expressed between luminal A tumors in COCA cluster 1 and COCA cluster 4 show the importance of their over expression in cancer cell survival. The luminal breast cancer cell line MCF-7 was transfected with miRNA mimics (20 nM) and assayed for cell proliferation (Ki67) (**a**); apoptosis (cleaved PARP (cPARP)) (**b**); estrogen receptor (ER) levels (**c**); phosphorylated AKT (p-AKT) levels (**d**); cell viability (**e**), 72 hours after transfection. Cell viability data are from two replicate experiments with *error bars* showing standard deviations. **a**-**d** Values ±2 × standard deviation (SD) were considered significant, corresponding to a threshold of |1.96| (see “Supplementary methods”). For the cell viability measures (**e**), values ±2 × SD were considered significant. The *error bars* for the negative controls (*miR neg ctrl*) show SD from four (**a**-**d**) or eight (**e**) replicates. (PDF 279 kb)

Additional file 9: Pathway enrichment map of genes correlated with miRNAs differentially expressed between luminal A tumors in COCA cluster 1 and COCA cluster 4 and upregulated in luminal A tumors in COCA cluster 4. A *blue line* connects any two pathways when there are more than five genes in common between them (exact number indicated). Ingenuity Pathway Analysis (IPA) was used to identify enriched pathways among the upregulated genes. (PDF 58 kb)

Additional file 10: Six proteins differentially expressed between luminal A samples in COCA cluster 1 versus COCA cluster 4. (PDF 771 kb)

## Abbreviations

CAAI: Complex arm aberration index
CNA: Copy number aberration
COCA: Cluster-of-clusters analysis
cPARP: Cleaved poly (ADP-ribose) polymerase
DBCG: Danish Breast Cancer Cooperative Group
ER: Estrogen receptor
FDR: False discovery rate
GEO: Gene Expression Omnibus
HER2: Human epidermal growth factor receptor 2
HR MAS MRS: High-resolution magic-angle spinning magnetic resonance spectroscopy
iClustering: Integrative clustering
IHC: Immunohistochemical assessment
IntClust: Integrative clusters
IPA: Ingenuity Pathway Analysis
IPL: Inferred pathway level
METABRIC: Molecular Taxonomy of Breast Cancer International Consortium
Micma: Oslo Micrometastasis cohort
miRNA: microRNA
NSC: Nearest shrunken centroid
p-AKT: phosphorylated AKT
PARADIGM: Pathway recognition algorithm using data integration on genomic models
PART: Partitioning algorithm using recursive thresholding
PR: Progesterone receptor
RIN: RNA integrity number
RPPA: Reverse-phase protein array
SD: Standard deviation
SNP: single nucleotide polymorphism
TCGA: The Cancer Genome Atlas
