# Supplementary material for: Integrative clustering reveals a novel split in the luminal A subtype of breast cancer with impact on outcome
Source: Breast Cancer Res. 2017 Mar 29;19:44. doi: 10.1186/s13058-017-0812-y (PMC5372339; doi:10.1186/s13058-017-0812-y)
Supplement: Supplementary file 2 — a Supplementary methods. b Summary of clinicopathological properties of the 425 primary breast tumors in the Oslo2 cohort. (PDF 137 kb) [file 13058_2017_812_MOESM2_ESM.pdf]

# Additional file 2

### Additional file 2a. Supplementary methods

#### miRNA and mRNA differential expression in luminal A tumors of COCA cluster 1 versus COCA cluster 4

A wilcoxon rank-sum test was applied to identify miRNAs that were differentially expressed between luminal A tumors of COCA cluster 1 vs. COCA cluster 4. The resulting p-values were corrected for multiple comparisons using the Benjamini-Hochberg FDR [1]. Differentially expressed miRNAs were defined as those with a corrected p-value < 0.01 and with an absolute $\log_2$ fold change >1. Fold change for each miRNAs was calculated as $-\log_2$ of the (non-transformed median of luminal A COCA 1)/(non-transformed median of luminal A COCA 4).

The expression of the 71 miRNAs was correlated to all genes using Spearman correlation and genes with correlation >|0.4| were retained, resulting in 1808 unique genes (only the expression of luminal A tumors of COCA cluster 1 and COCA cluster 4 were considered). To further test which of these 1808 genes were also differentially expressed between luminal A tumors of COCA cluster 1 vs. COCA cluster 4, a t-test was applied. Genes with a Benjamini-Hochberg corrected p-value <0.05 were further considered, resulting in 1323 genes of which 473 genes were upregulated in COCA cluster 1 compared to COCA cluster 4 and 850 genes were upregulated in COCA cluster 4 compared to COCA cluster 1 (considering the sign of the fold change). These two gene lists were further taken separately into the core analysis application of QIAGEN's Ingenuity® Pathway Analysis (IPA®, QIAGEN Redwood City, www.qiagen.com/ingenuity) where canonical pathways enriched among the genes in each list were identified using a Fisher's exact test. Only pathways with a Benjamini-Hochberg corrected p-value <0.05 were further considered.

#### miRNA functional assays

MCF-7 cells [2, 3] were purchased from Interlab Cell Line Collection (ICLC, Genova, Italy) and cultured in DMEM (1 g/l glucose; Sigma-Aldrich, St Louis, MO, USA) supplemented with 10% fetal bovine serum (FBS), 2 mM L-glutamine and 1% penicillin/streptomycin. For the functional assays, cells were transfected with miRIDIAN miRNA mimics (Dharmacon, Lafayette, CO, USA) at 20 nM concentration in 384-well plates using SilentFect (Bio-Rad Laboratories, Hercules, CA, USA) as described previously [4, 5]. Four different negative

controls were considered, two from Dharmacon (replicates merged and represented as "mir neg ctrl #1") and two from Ambion (Thermo Fisher Scientific Inc., Waltham, MA, USA; replicates merged and represented as "mir neg ctrl #2"). After 72 hours of incubation, cell viability was assayed by CellTiter-GLO cell viability assay (Promega Corp., Madison, WI, USA). The results were Loess normalized [6] and $\log_2$-transformed. Values ±2 × standard deviation (SD), were considered as significant.

For protein lysate microarray analysis, cells were lysed 72 hours after transfection and printed on nitrocellulosecoated microarray FAST™ slides (Whatman Inc., Florham Park, NJ, USA). Ki67, cleaved PARP (cPARP), ER and phosphorylated AKT (p-AKT) were detected by staining the slides with Ki67 antibody (#M7240, Dako, Glostrup, Denmark), cPARP antibody (#ab32064, Abcam, Cambridge, UK), ERalpha antibody (Ab-15; Labvision Corp, Fremont, CA) and p-AKT(S473) antibody (#9271, Cell Signaling Technology Inc., Danvers, MA, USA), respectively, followed by exposure to Alexa Fluor 680-tagged secondary antibodies (Invitrogen Inc.). For total protein measurement, the arrays were stained with Sypro Ruby Blot solution (Invitrogen Inc.). The slides were scanned with Tecan LS400 (Tecan Inc., Durham, NC, USA) microarray scanner and Odyssey Licor IR-scanner (LI-COR Biosciences, Lincoln, NE, USA) to detect the Sypro, Ki67, cPARP, ER and p-AKT signals. Array-Pro Analyzer microarray analysis software (Median Cybernetics Inc., Bethesda, MD, USA) was used for analyzing the data. The lysate microarray data were $\log_2$-transformed and converted into z-scores by subtracting the mean of the whole screen and dividing by the standard deviation of the whole screen. Values ±2 × SD were considered as significant, which corresponded to a threshold of $|1.96|$.

**Associating the luminal A split to COCA input levels**

To assess if the luminal A samples in the two clusters were different with respect to COCA input levels other than miRNA clusters (i.e. CAAI subgroups, iClusters, metabolic clusters, PARADIGM clusters and RPPA subtypes), Chi-squared association tests were used with a significance threshold of p-value <0.05.

**Associating miRNAs and proteins differentially expressed between luminal A tumors of COCA cluster 1 versus COCA cluster 4**

To test which proteins (from RPPA data) were differentially expressed between luminal A tumors of COCA cluster 1 versus COCA cluster 4, t-tests were used. Proteins with a Benjamini-Hochberg corrected p-value <0.05 were further considered.

To obtain predicted target genes of the miRNAs differentially expressed between the two luminal A subgroups, the list of 71 miRNAs were taken into the microRNA Target Filter application filter of IPA. Here, *in silico* predicted target genes were identified using the TargetScan Human prediction algorithm [7]. The list of predicted target genes was then overlaid with the list of the six proteins differentially expressed between the luminal A subgroups. To assess the correlation in expression between miRNA and proteins, Spearman correlation was calculated using expression data from all luminal A tumors with both miRNA and protein expression available (n = 123).

**Assessing the luminal A split in independent breast cancer cohorts**

Four breast cancer cohorts with available miRNA expression and follow-up data were used to assess if the differentially expressed miRNAs between the two luminal A groups of COCA cluster 1 and COCA cluster 4 would split luminal A tumors in independent cohorts and if any prognostic difference was found in the split. The luminal A subtype was scored according to the PAM50 classification [8]. miRNA sequencing data (Illumina, San Diego, CA, USA) from The Cancer Genome Atlas (TCGA) [9] were downloaded from Broad GDAC Firehose (accessed 15 January 2014) and follow-up data (overall survival) were downloaded from the TCGA Data Portal (accessed 24 February 2016). For the METABRIC cohort, miRNA expression data (Agilent Technologies) was available from [10], deposited in the European Genome-Phenome Archive, www.ebi.ac.uk/ega, accession number EGAS00000000122 and follow-up data were available from [11]. For the Danish Breast Cancer Cooperative Group (DBCG) cohort [12, 13], miRNA expression data (Agilent Technologies) were available from [14] and follow-up data (any recurrence) from [15]. For the Oslo Micrometastasis (Micma) cohort [16], miRNA expression (Agilent Technologies) was available from [17] and follow-up data (any recurrence) available from [18]. Altogether, 56 of the 71 differentially expressed miRNAs were available in the TCGA, DBCG and Micma cohorts, and 68 miRNAs were available from the METABRIC cohort and thus considered in the cluster analysis. Luminal A patient clusters were derived using Pearson correlation as distance measure and complete linkage (as was used in the original miRNA clusters found in the Oslo2 cohort), and the two main patient clusters were further considered (three TCGA samples and 25 METABRIC samples forming "outlier" clusters were not considered). Kaplan-Meier survival analyses and log-rank tests were performed using the R package 'survival'. For the METABRIC cohort the log-rank p-value was adjusted for hospital site and for the DBCG cohort the log-rank p-value was adjusted for radiation therapy and lymph node status.

## References


1. Benjamini Y, Hochberg Y: **Controlling the False Discovery Rate - a Practical and Powerful Approach to Multiple Testing**. *Journal of the Royal Statistical Society Series B-Methodological* 1995, **57**(1):289 - 300.
2. Brooks SC, Locke ER, Soule HD: **Estrogen Receptor in a Human Cell Line (MCF-7) from Breast Carcinoma**. *Journal of Biological Chemistry* 1973, **248**(17):6251-6253.
3. Soule H, Vazguez J, Long A, Albert S, Brennan M: **A Human Cell Line From a Pleural Effusion Derived From a Breast Carcinoma**. *J Natl Cancer Inst* 1973, **51**(5):1409-1416.
4. Leivonen SK, Makela R, Ostling P, Kohonen P, Haapa-Paananen S, Kleivi K, Enerly E, Aakula A, Hellstrom K, Sahlberg N *et al*: **Protein lysate microarray analysis to identify microRNAs regulating estrogen receptor signaling in breast cancer cell lines**. *Oncogene* 2009, **28**(44):3926-3936.
5. Leivonen SK, Sahlberg KK, Makela R, Due EU, Kallioniemi O, Borresen-Dale AL, Perala M: **High-throughput screens identify microRNAs essential for HER2 positive breast cancer cell growth**. *Mol Oncol* 2014, **8**(1):93-104.
6. Boutros M, Bras L, Huber W: **Analysis of cell-based RNAi screens**. *Genome Biology* 2006, **7**(7):R66.
7. Lewis BP, Burge CB, Bartel DP: **Conserved Seed Pairing, Often Flanked by Adenosines, Indicates that Thousands of Human Genes are MicroRNA Targets**. *Cell* 2005, **120**(1):15-20.
8. Parker JS, Mullins M, Cheang MCU, Leung S, Voduc D, Vickery T, Davies S, Fauron C, He X, Hu Z *et al*: **Supervised Risk Predictor of Breast Cancer Based on Intrinsic Subtypes**. *Journal of Clinical Oncology* 2009, **27**(8):1160-1167.
9. The Cancer Genome Atlas Network: **Comprehensive molecular portraits of human breast tumours**. *Nature* 2012, **490**(7418):61-70.
10. Dvinge H, Git A, Graf S, Salmon-Divon M, Curtis C, Sottoriva A, Zhao Y, Hirst M, Armisen J, Miska EA *et al*: **The shaping and functional consequences of the microRNA landscape in breast cancer**. *Nature* 2013, **497**:378-382.
11. Curtis C, Shah SP, Chin S-F, Turashvili G, Rueda OM, Dunning MJ, Speed D, Lynch AG, Samarajiwa S, Yuan Y *et al*: **The genomic and transcriptomic architecture of 2,000 breast tumours reveals novel subgroups**. *Nature* 2012, **486**(7403):346-352.
12. Overgaard M, Hansen PS, Overgaard J, Rose C, Andersson M, Bach F, Kjaer M, Gadeberg CC, Mouridsen HT, Jensen M-B *et al*: **Postoperative Radiotherapy in High-Risk Premenopausal Women with Breast Cancer Who Receive Adjuvant Chemotherapy**. *New England Journal of Medicine* 1997, **337**(14):949-955.
13. Overgaard M, Jensen M-B, Overgaard J, Hansen PS, Rose C, Andersson M, Kamby C, Kjaer M, Gadeberg CC, Rasmussen BB *et al*: **Postoperative radiotherapy in high-risk postmenopausal breast-cancer patients given adjuvant tamoxifen: Danish Breast Cancer Cooperative Group DBCG 82c randomised trial**. *The Lancet* 1999, **353**(9165):1641-1648.
14. Aure MR, Steinfeld I, Baumbusch LO, Liestøl K, Lipson D, Nyberg S, Naume B, Sahlberg KK, Kristensen VN, Børresen-Dale A-L *et al*: **Identifying In-*Trans* Process Associated Genes in Breast Cancer by Integrated Analysis of Copy Number and Expression Data**. *PLoS ONE* 2013, **8**(1):e53014.
15. Tramm T, Mohammed H, Myhre S, Kyndi M, Alsner J, Børresen-Dale A-L, Sørlie T, Frigessi A, Overgaard J: **Development and Validation of a Gene Profile Predicting Benefit of Postmastectomy Radiotherapy in Patients with High-Risk Breast Cancer: A Study of Gene Expression in the DBCG82bc Cohort**. *American Association for Cancer Research* 2014, **20**(20):5272-5280.

16. Naume B, Borgen E, Kvalheim G, Kåresen R, Qvist H, Sauer T, Kumar T, Nesland JM: **Detection of Isolated Tumor Cells in Bone Marrow in Early-Stage Breast Carcinoma Patients**. *Clinical Cancer Research* 2001, **7**(12):4122-4129.
17. Enerly E, Steinfeld I, Kleivi K, Leivonen S-K, Aure MR, Russnes HG, Rønneberg JA, Johnsen H, Navon R, Rødland E *et al*: **miRNA-mRNA Integrated Analysis Reveals Roles for miRNAs in Primary Breast Tumors**. *PLoS ONE* 2011, **6**(2):e16915.
18. Naume B, Zhao X, Synnestvedt M, Borgen E, Russnes HG, Lingjærde OC, Strømberg M, Wiedswang G, Kvalheim G, Kåresen R *et al*: **Presence of bone marrow micrometastasis is associated with different recurrence risk within molecular subtypes of breast cancer**. *Molecular Oncology* 2007, **1**(2):160-171.

| Additional file 2b. Clinicopathological data of the Oslo2 cohort | | | |
|---|---|---|---|
| | | | |
| | | **Number of patients** | **% (Excluding missing)** |
| **All** | | 425 | 100 |
| | | | |
| **ER** | Positive | 313 | 80.3 |
| | Negative | 77 | 19.7 |
| | Missing | 35 | |
| | | | |
| **PR** | Positive | 266 | 68.4 |
| | Negative | 123 | 31.6 |
| | Missing | 36 | |
| | | | |
| **HER2** | Positive | 47 | 12.3 |
| | Negative | 336 | 87.7 |
| | Missing | 42 | |
| | | | |
| | | | |
| **Grade** | I | 59 | 14.8 |
| | II | 167 | 42.0 |
| | III | 172 | 43.2 |
| | Missing | 27 | |
| | | | |
| ***TP53*** | Wild type | 255 | 67.5 |
| | Mutant | 123 | 32.5 |
| | Missing | 47 | |
| | | | |
| ***PIK3CA*** | Wild type | 237 | 69.7 |
| | Mutant | 103 | 30.3 |
| | Missing | 85 | |
| | | | |
| **Nodal status** | LN met present* | 158 | 39.6 |
| | LN met not present (N0) | 241 | 60.4 |
| | Missing | 26 | |
| | | | |
| **Age (years)** | <50 | 107 | 26.9 |
| | 50-70 | 234 | 58.8 |
| | >70 | 57 | 14.3 |
| | Missing | 27 | |
| | | | |
| | | | |
| **Tumor size (mm)** | <10 | 29 | 7.3 |
| | 10-20 | 186 | 46.5 |
| | >20 | 185 | 46.3 |

| | | | |
|---|---|---|---|
| | Missing | 25 | |
| | | | |
| **Histology** | DCIS | 10 | 2.5 |
| | Ductal | 326 | 81.9 |
| | Lobular | 40 | 10.1 |
| | Medullary | 1 | 0.3 |
| | Metaplastic | 1 | 0.3 |
| | Mix ductal lobular | 2 | 0.5 |
| | Mixed | 4 | 1.0 |
| | Mucinous | 6 | 1.5 |
| | PapillaryCIS | 1 | 0.3 |
| | Tubular | 5 | 1.3 |
| | Tubulolobular | 2 | 0.5 |
| | Missing | 27 | |
| | | | |
| | * Sum of N+, N1, N1(mi), N1a, N2 and N3 | | |
