# Supplementary figures and images for: Integrative clustering reveals a novel split in the luminal A subtype of breast cancer with impact on outcome
Source: Breast Cancer Res. 2017 Mar 29;19:44. doi: 10.1186/s13058-017-0812-y (PMC5372339; doi:10.1186/s13058-017-0812-y)

Additional file 3

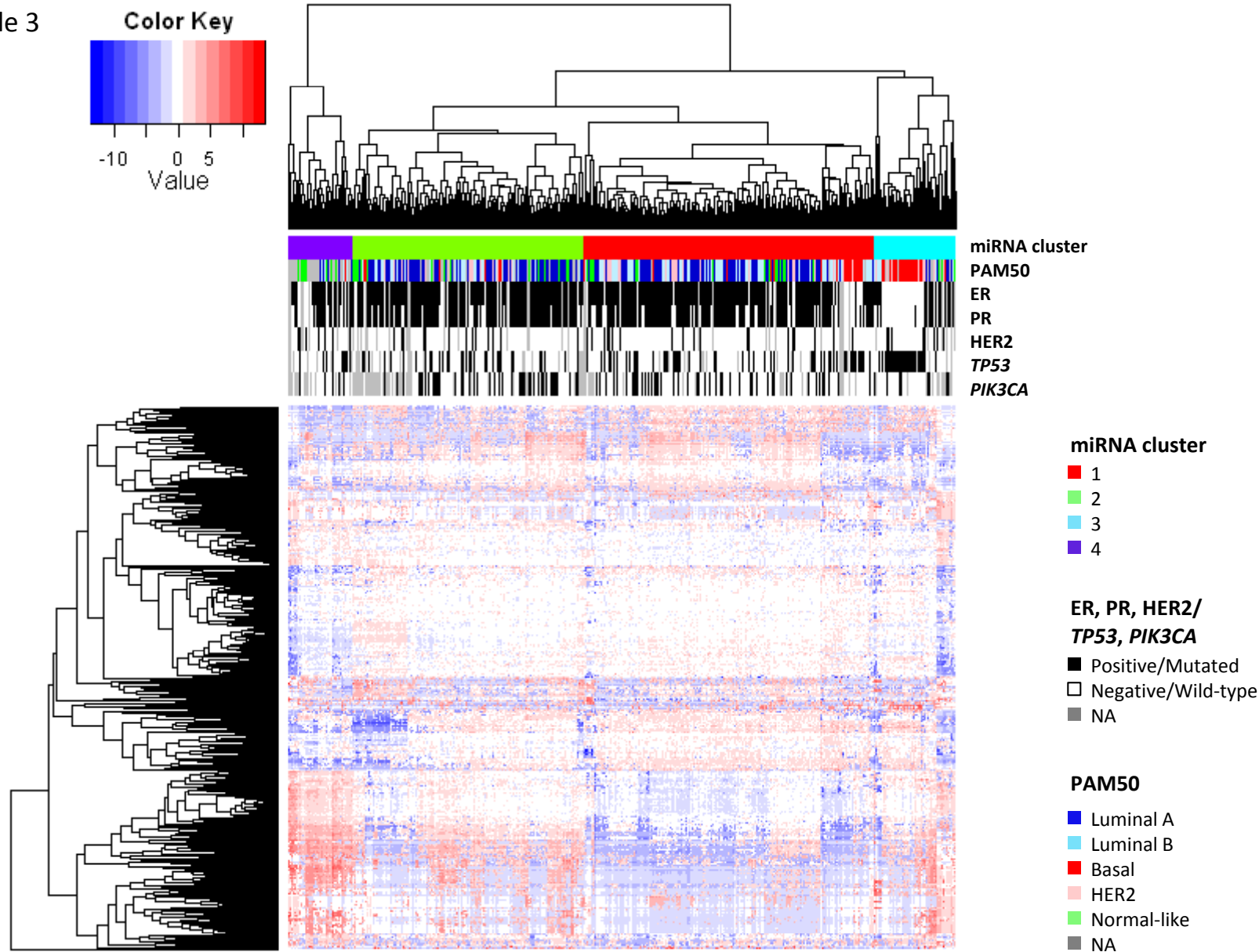

Supplement: Supplementary file 3 — Four miRNA patient clusters (1‒4) derived from clustering the expression of 421 miRNAs using Pearson correlation and complete linkage. The PART algorithm was used to identify clusters [23]. (PDF 136 kb) [file 13058_2017_812_MOESM3_ESM.pdf]

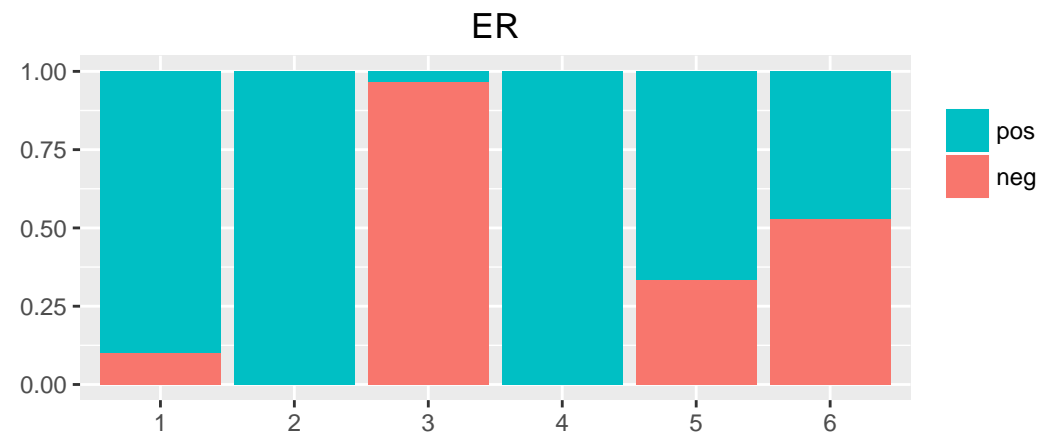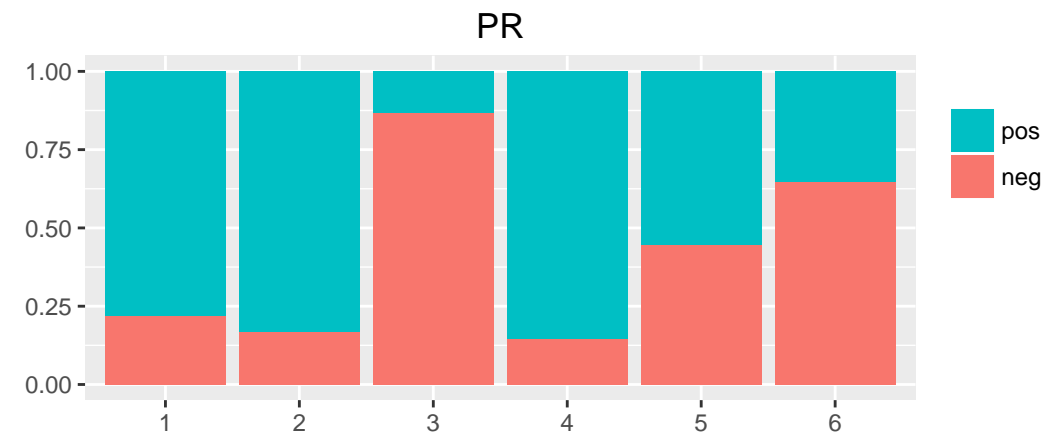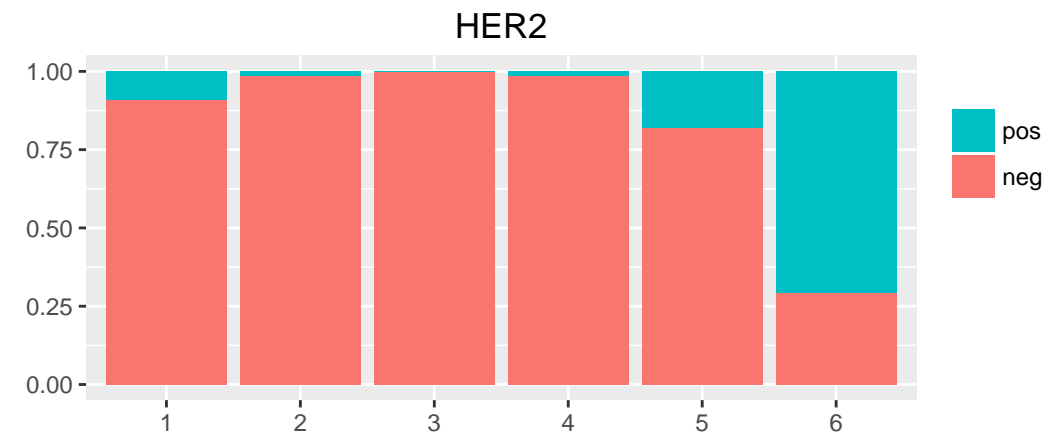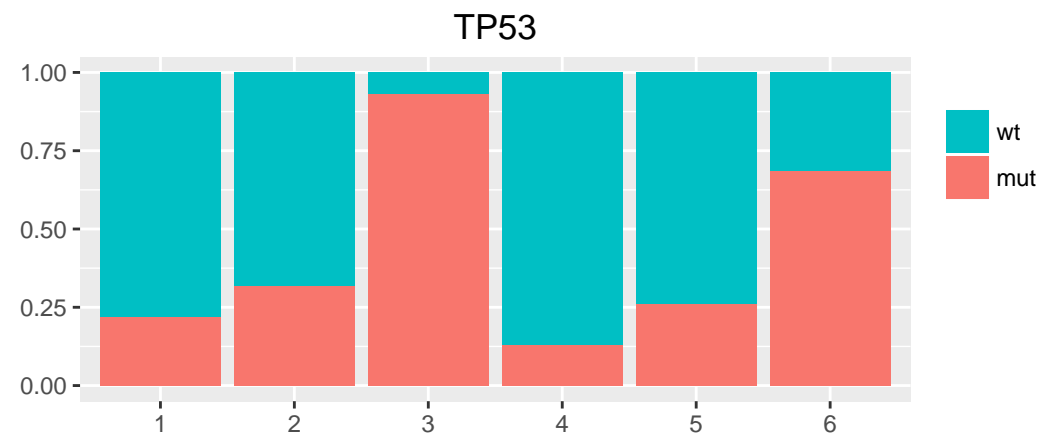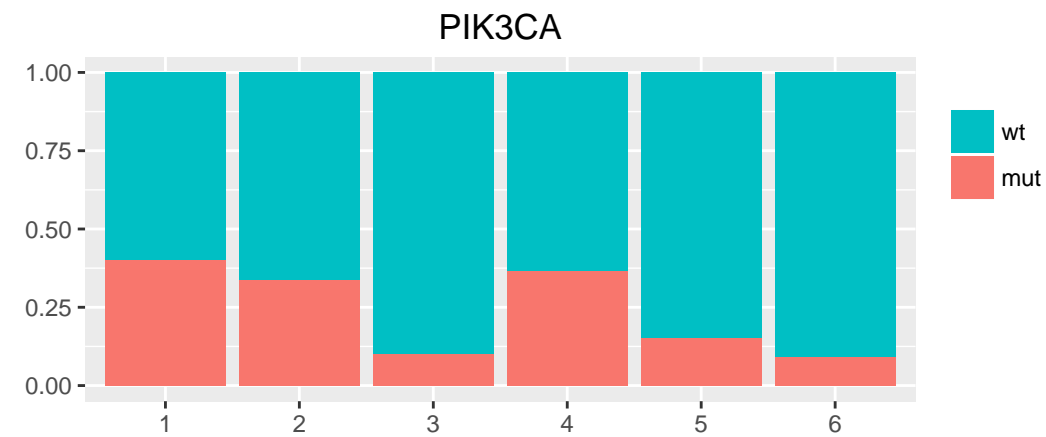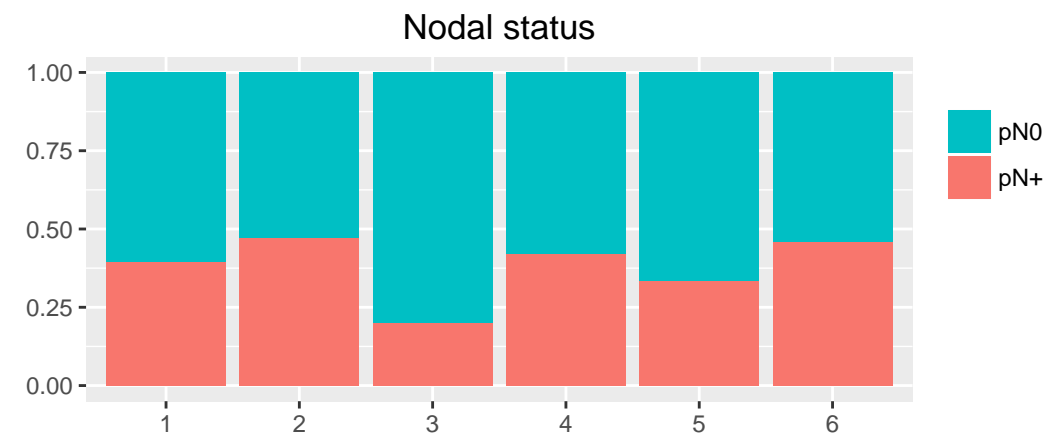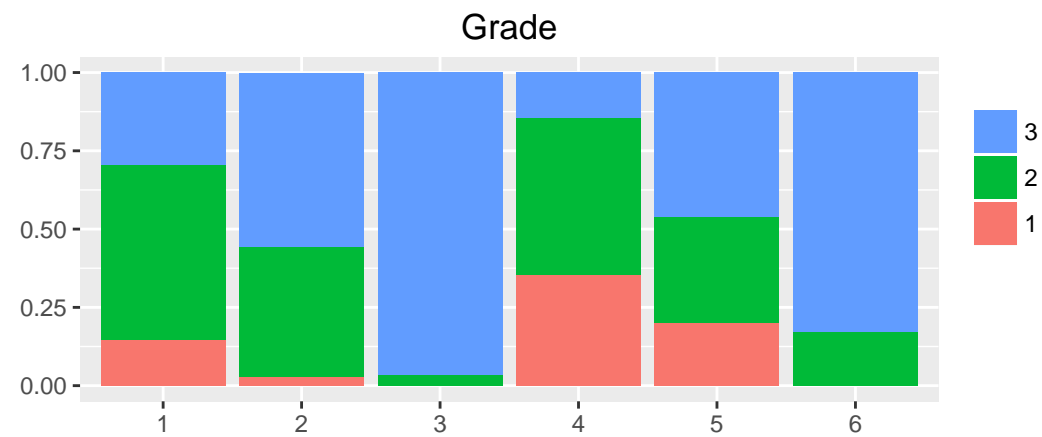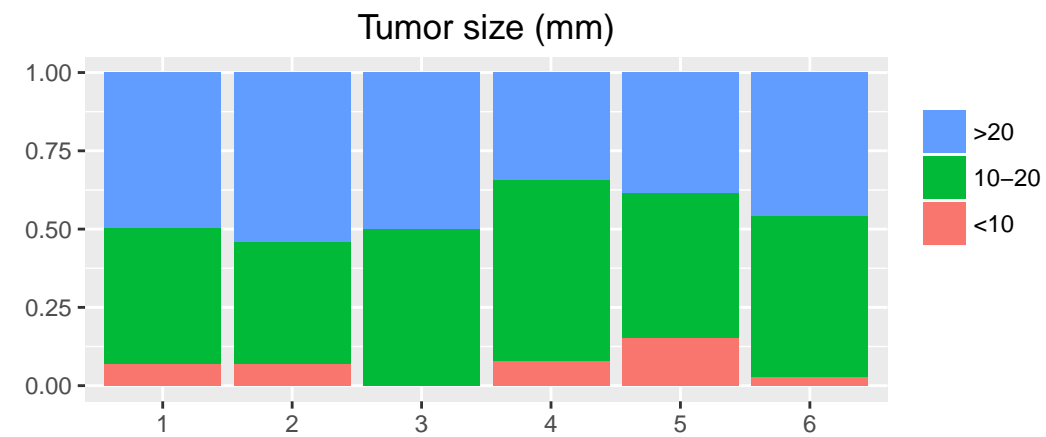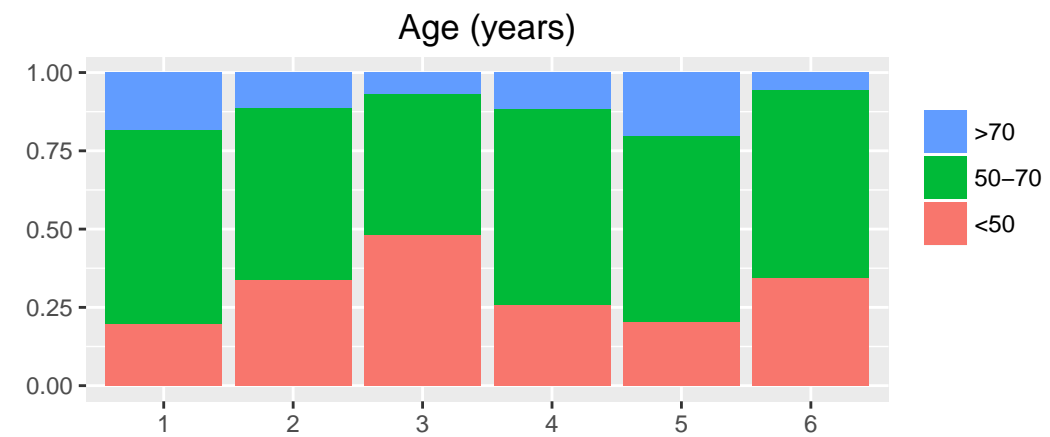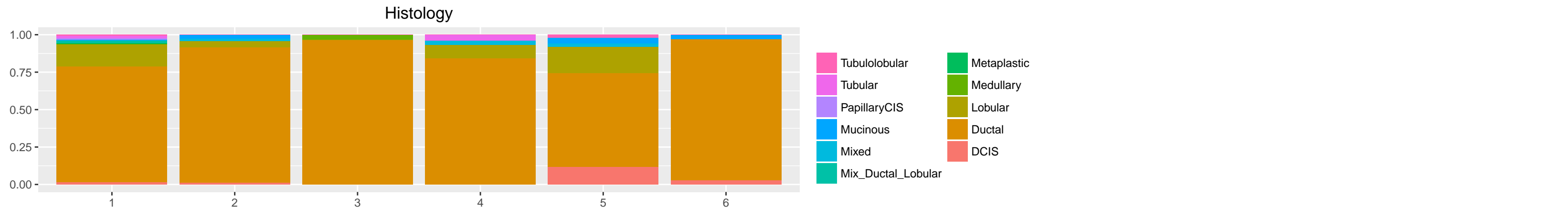

Supplement: Supplementary file 7 — Clinical and molecular distribution in the six COCA clusters. (PDF 7 kb) [file 13058_2017_812_MOESM7_ESM.pdf]

## (a) Proliferation

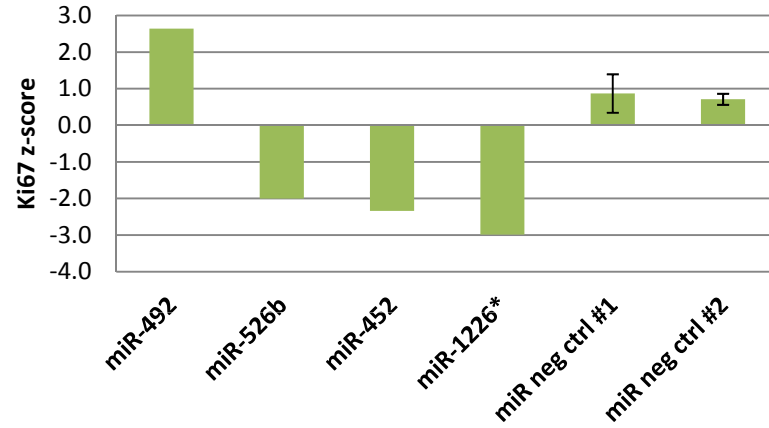

## (b) Apoptosis

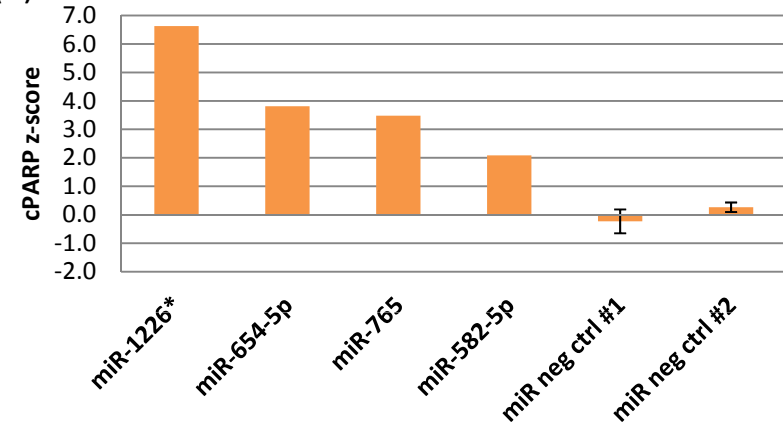

## (c) Estrogen receptor

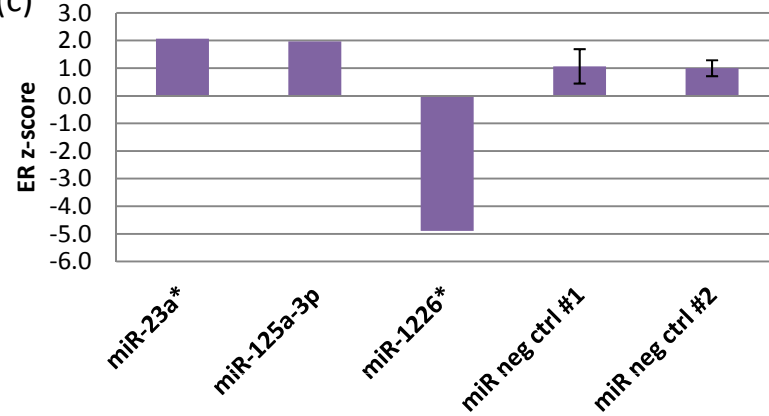

## (d) p-AKT

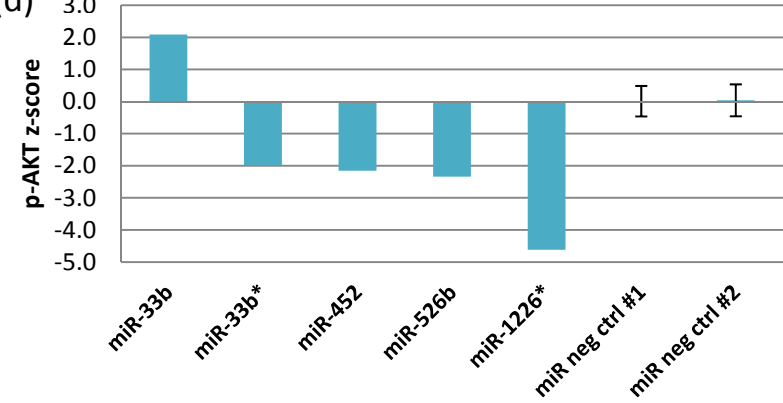

## (e) Cell viability

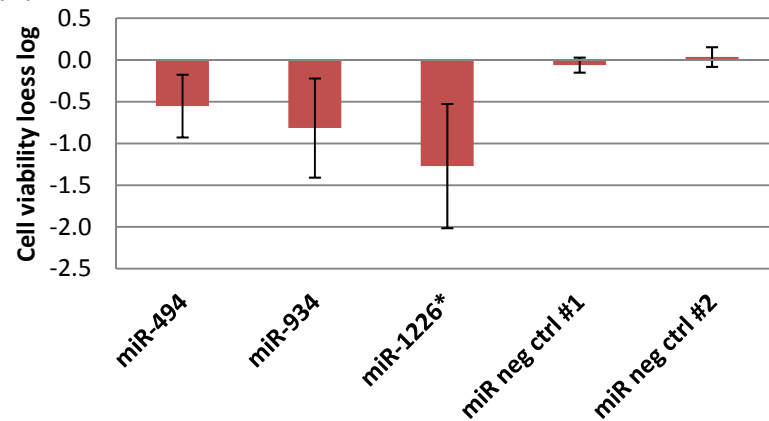

Supplement: Supplementary file 8 — Functional studies of miRNAs differentially expressed between luminal A tumors in COCA cluster 1 and COCA cluster 4 show the importance of their over expression in cancer cell survival. The luminal breast cancer cell line MCF-7 was transfected with miRNA mimics (20 nM) and assayed for cell proliferation (Ki67) (a); apoptosis (cleaved PARP (cPARP)) (b); estrogen receptor (ER) levels (c); phosphorylated AKT (p-AKT) levels (d); cell viability (e), 72 hours after transfection. Cell viability data are from two replicate experiments with error bars showing standard deviations. a-d Values ±2 × standard deviation (SD) were considered significant, corresponding to a threshold of |1.96| (see “Supplementary methods”). For the cell viability measures (e), values ±2 × SD were considered significant. The error bars for the negative controls (miR neg ctrl) show SD from four (a-d) or eight (e) replicates. (PDF 279 kb) [file 13058_2017_812_MOESM8_ESM.pdf]

## Additional file 9

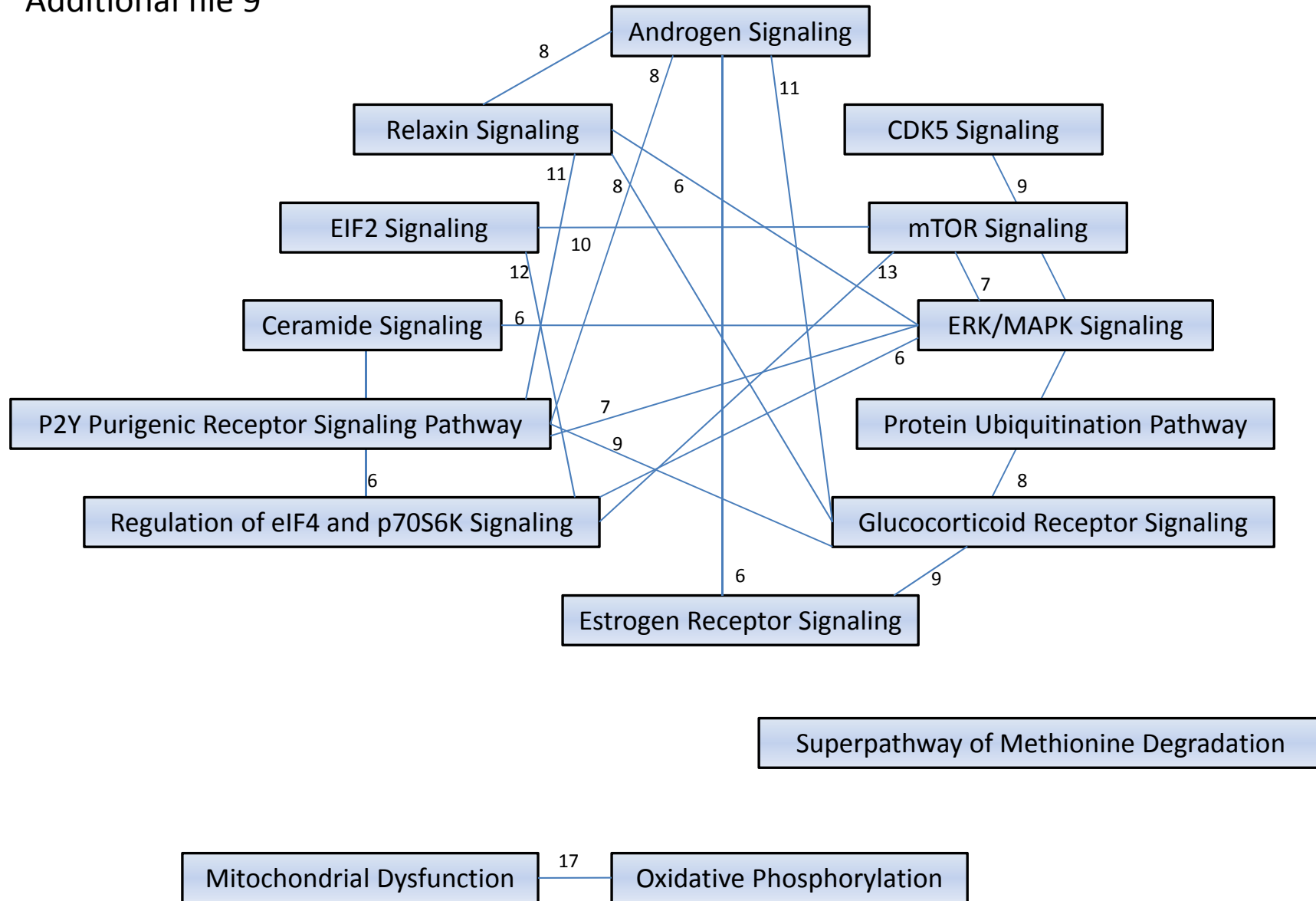

Supplement: Supplementary file 9 — Pathway enrichment map of genes correlated with miRNAs differentially expressed between luminal A tumors in COCA cluster 1 and COCA cluster 4 and upregulated in luminal A tumors in COCA cluster 4. A blue line connects any two pathways when there are more than five genes in common between them (exact number indicated). Ingenuity Pathway Analysis (IPA) was used to identify enriched pathways among the upregulated genes. (PDF 58 kb) [file 13058_2017_812_MOESM9_ESM.pdf]

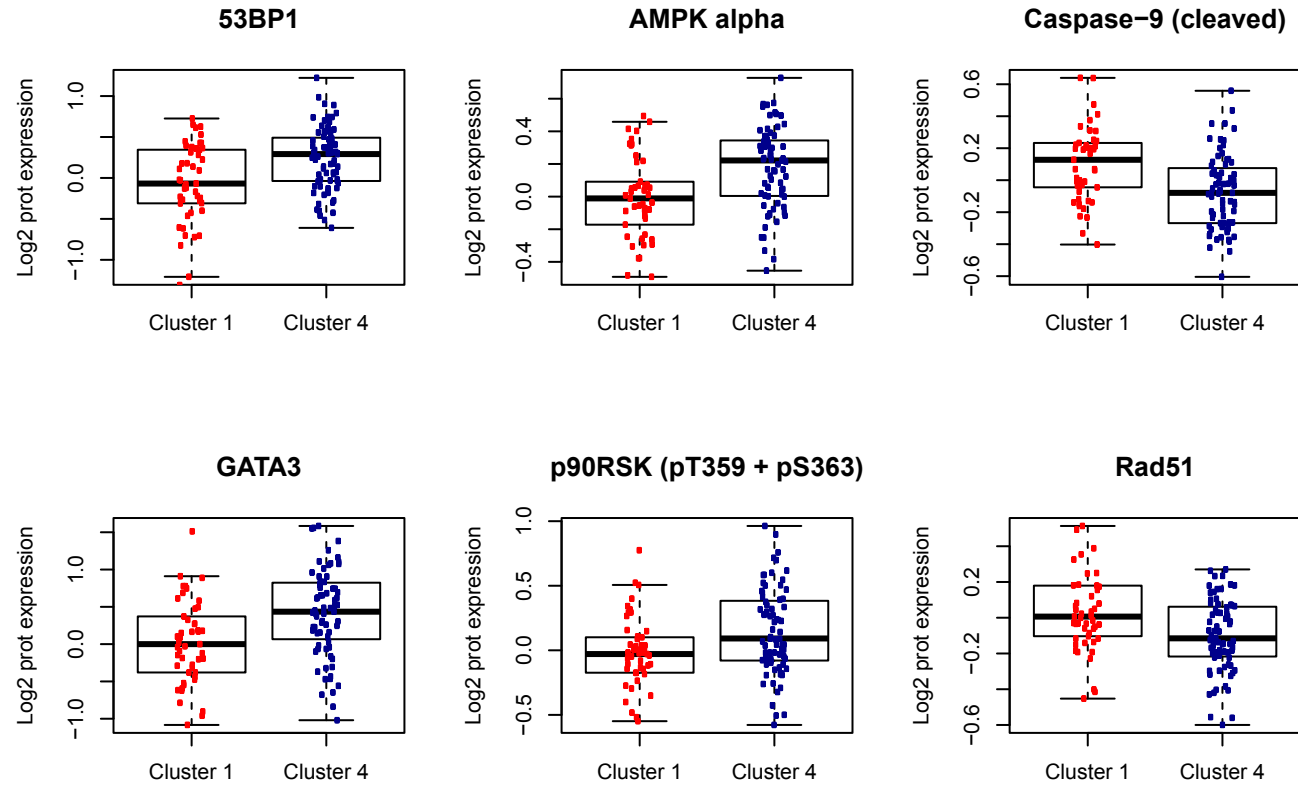

Supplement: Supplementary file 10 — Six proteins differentially expressed between luminal A samples in COCA cluster 1 versus COCA cluster 4. (PDF 771 kb) [file 13058_2017_812_MOESM10_ESM.pdf]
