# Supplementary material for: Integrative clustering reveals a novel split in the luminal A subtype of breast cancer with impact on outcome
Source: Breast Cancer Res. 2017 Mar 29;19:44. doi: 10.1186/s13058-017-0812-y (PMC5372339; doi:10.1186/s13058-017-0812-y)

Additional file 5

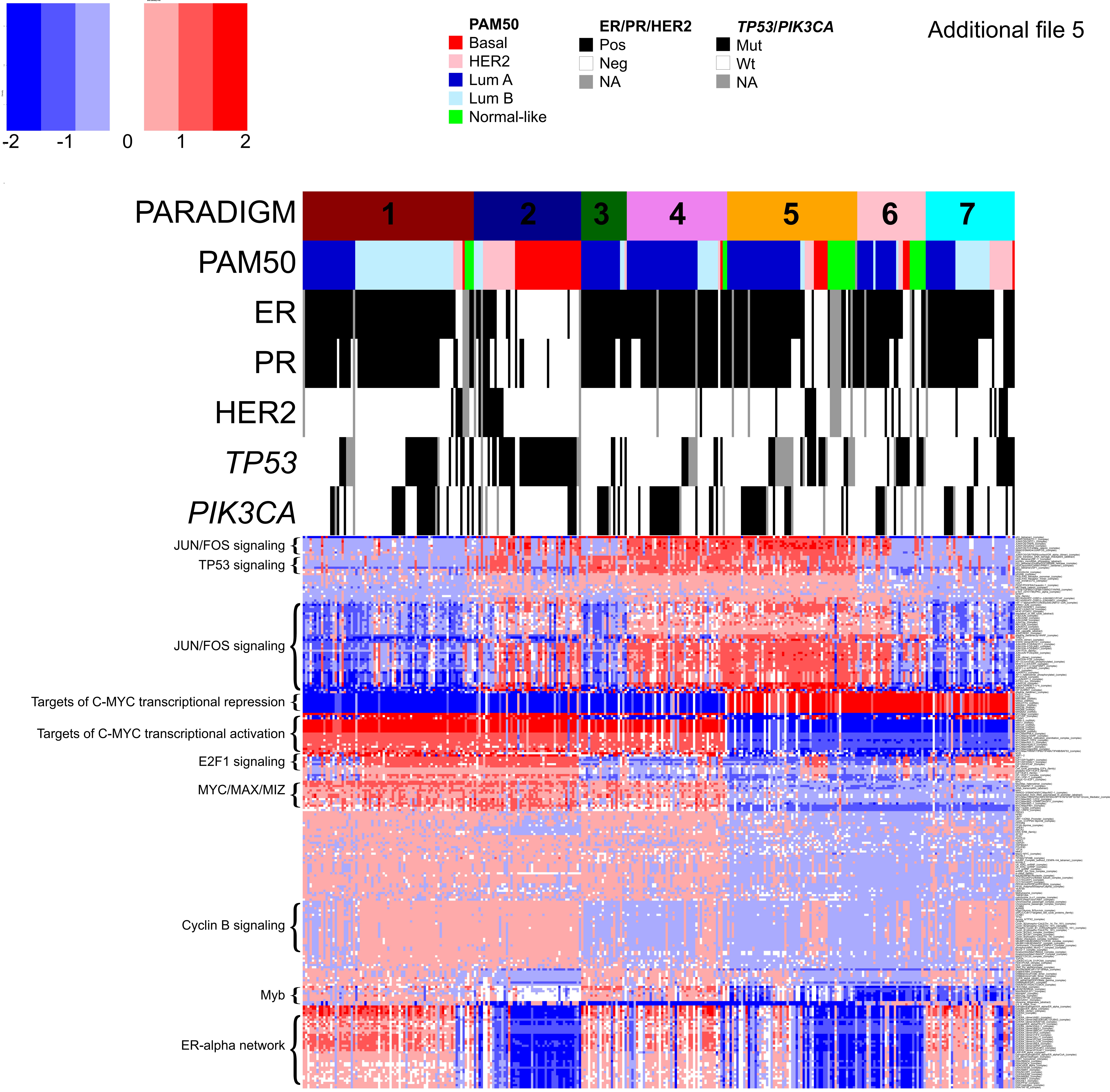

Supplement: Supplementary file 5 — Oslo2 tumors sorted according to membership of each of seven PARADIGM clusters (columns) with heatmap representation of the top 253 most deregulated pathway entities (IPLs) across the clusters (rows; filtering out IPLs with activity -0.25 > x < 0.25). IPL name details can be seen by zooming in. (PDF 2274 kb) [file 13058_2017_812_MOESM5_ESM.pdf]
